# Fungistatic Effect of Phthalide Lactones on *Rhodotorula mucilaginosa*

**DOI:** 10.3390/molecules28145423

**Published:** 2023-07-15

**Authors:** Joanna Gach, Teresa Olejniczak, Jakub Pannek, Filip Boratyński

**Affiliations:** Department of Food Chemistry and Biocatalysis, Wrocław University of Environmental and Life Sciences, Norwida 25, 50-375 Wrocław, Poland; jpannek@gmail.com (J.P.); filip.boratynski@upwr.edu.pl (F.B.)

**Keywords:** broth microdilution, biomass determination, synergistic effect, fatty acid methyl esters, carotenoids

## Abstract

Currently, there is an increasing number of cases of fungal infections caused by opportunistic strains of the yeast *Rhodotorula mucilaginosa*, mainly in immunocompromised patients during hospitalization. The excessive use of antibiotics and azole compounds increases the risk of resistance to microorganisms. A new alternative to these drugs may be synthetic phthalide lactones with a structure identical to or similar to the natural ones found in celery plants, which show low toxicity and relatively high fungistatic activity. In the present study, the fungistatic activity of seven phthalide lactones was determined against *R. mucilaginosa* IHEM 18459. We showed that 3-*n*-butylidenephthalide, the most potent compound selected in the microdilution test, caused a dose-dependent decrease in dry yeast biomass. Phthalide accumulated in yeast cells and contributed to an increase in reactive oxygen species content. The synergistic effect of fluconazole resulted in a reduction in the azole concentration required for yeast inhibition. We observed changes in the color of the yeast cultures; thus, we conducted experiments to prove that the carotenoid profile was altered. The addition of lactones also triggered a decline in fatty acid methyl esters.

## 1. Introduction

*Rhodotorula mucilaginosa* is a saprophytic eukaryote ubiquitous in the ecological environment. Numerous strains of *R. mucilaginosa* are being tested for their biotechnological importance in the production of carotenoids, lipids, enzymes, and other functional bioproducts, using cheap agricultural waste [1,2]. *R. mucilaginosa* can be isolated from food, mainly cheese, dairy products, sausages, and fruit juices [3,4]. There is a growing concern that food may be an underestimated source of environmental pathogens [5]. Moreover, for many years, we have observed some pathogenic strains that cause local mycosis of the skin in critically ill patients and those taking immunosuppressive medications [6]. There is a slow but alarming increase in the number of bloodstream infections associated with the use of central venous catheters. *Rhodotorula* infections of the eyes, meninges, prosthetic joints, and peritoneum occur less frequently. Many have no documented relationship with central venous catheter use or immunosuppression. These infections occur mainly in patients receiving corticosteroids, cytotoxic drugs, and broad-spectrum antibiotics [7,8,9,10].

*R. mucilaginosa* infections are treated similarly to other pathogenic yeast infections with drugs from the azole group (fluconazole, itraconazole, and ketoconazole), 5-fluorocytosine, and amphotericin. Several studies reported on the resistance of microorganisms to commonly used antibiotics. The so-called multidrug-resistant microorganisms include some *Candida*, *Aspergillus*, and *Cryptococcus* strains resistant to azole drugs, and methicillin-resistant *Staphylococcus aureus* [11,12].

In addition to the search for new drugs, one of the methods to tackle the problem of resistance is to use the synergistic effect. This phenomenon has been studied in various strains of *Candida*, including drug-resistant strains. In the case of *C. albicans*, a synergistic effect with fluconazole and 3-*n*-butylphthalide allowed for a 5–20-fold reduction in the doses [13,14,15]. The aforementioned 3-*n*-butylphthalide belongs to the group of phthalide lactones and is found in various celery plants: *Angelica archangelica* (angelica), *A. sinensis* (Chinese angelica), *Apium graveolens* (celery), *Cnidium officinale*, and *Levisticum officinale* (lovage) [16].

Phthalides have been studied for their antimicrobial properties against phytopathogens and species pathogenic to animals and humans [17]. For example, 3-*n*-butylidenephthalide is active against *Bacillus cereus*, *Escherichia coli*, *Listeria monocytogenes*, *Shigella flexneri*, *S. sonnei*, *Staphylococcus aureus*, *Vibrio harvei*, *V. parahaemolyticus*, and *V. vulnificus*. Some phthalides are active against *Mycobacterium bovis* and *M. tuberculosis*, which are the bacilli that cause tuberculosis in animals and humans, respectively [16,17,18].

**Figure 1:**
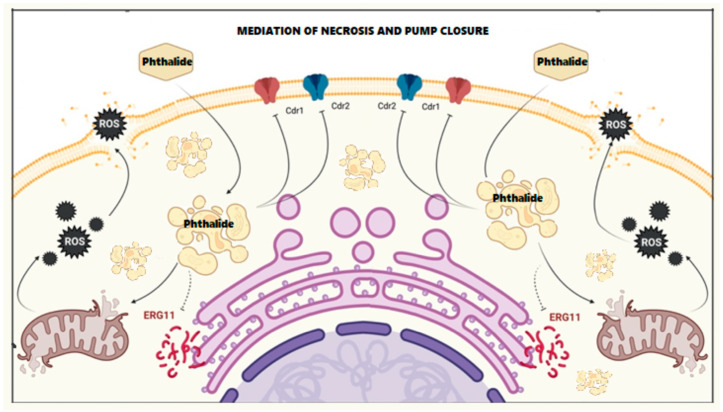
Mechanism of action of 3-*n*-butylphthalide on *C. albicans* (figure created in BioRender https://www.biorender.com/, accessed on 14 February 2023).

Gong et al. showed that the fungistatic 3-*n*-butylphthalide inhibited genes responsible for the biosynthesis of CDR1 and CDR2 transport pumps in *Candida albicans* strains isolated from patients, contributing to the accumulation of both lactones and azole compounds inside the cell [13]. The presence of the phthalide in the cytosol led to increased production of reactive oxygen species (ROS) by the mitochondria as demonstrated by measuring the mitochondrial membrane potential (Δψm). The accumulation of ROS resulted in the direct destruction of the mitochondrial cell membrane and the release of large amounts of ROS into the cytosol. The damaging effects of ROS include structural changes in DNA and RNA, peroxidation of membrane phospholipids (consisting of polyunsaturated fatty acids), and oxidation of proteins that perform numerous functions inside the cell. Figure 1 shows the mechanism of synergistic interactions between 3-*n*-butylphthalide and fluconazole. In our latest publication, we confirmed that this effect was shown not only by 3-*n*-butylphthalide but also by 3-*n*-butylidenephthalide and 3-*n*-butyl-4,5,6,7-tetrahydrophthalide [15].

To the best of our knowledge, no studies have described the effect of phthalide lactones on *Rhodotorula* strains. The main goal of our work was to deepen our knowledge of the action of this synthetic phthalide lactone with structures identical to or similar to natural compounds on yeast cells. In this study, we selected a strain of *R. mucilaginosa* that can colonize food products and constitutes a potential opportunistic strain. We were interested in the fact that the cultures of this microorganism lost their characteristic orange-red color in the presence of phthalide. We tested seven lactones against this strain. Owing to their low toxicity and, for most of them, natural occurrence, they can be used as yeast growth-inhibitory agents. We analyzed the effect of 3-*n*-butylidenephthalide on synergism with fluconazole, ROS content, carotenoids, fatty acid composition, and ergosterol production.

## 2. Results and Discussion

**Figure 2:**
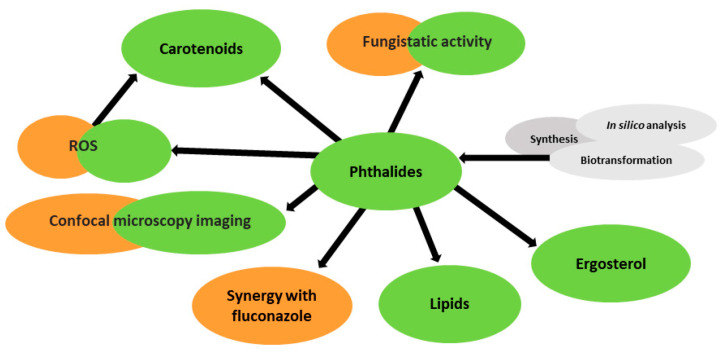
Schematic of the experiments.

In the first stage of this study, we synthesized and determined the fungistatic activities of seven lactones against *R. mucilaginosa* IHEM 18459. However, the main goal of this study was to explain the fungistatic effect of phthalide lactones. Figure 2 shows a diagram of the experiments conducted in this study.

In the next part of the study, we referred to the work of Gong et al. [13] and Krężel et al. [15] describing the effect of phthalide lactone on fluconazole-resistant *C. albicans* cells and compared it with the effect on *R. mucilaginosa* IHEM 18459 cells (Figure 2, orange).

Finally, we determined how 3-*n*-butylidenephthalide (**1**) affects the quantity and quality of carotenoids, fatty acid methyl ester (FAME), and ergosterol. We also visualized the cells using confocal microscopy (Figure 2, green). Other experiments, such as synthesis, biotransformations, and in silico analysis, unrelated to the elucidation of the fungistatic effect of phthalide lactones, are highlighted in gray (Figure 2, gray).

### 2.1. Synthesis of Phthalide Lactones

**Figure 3:**
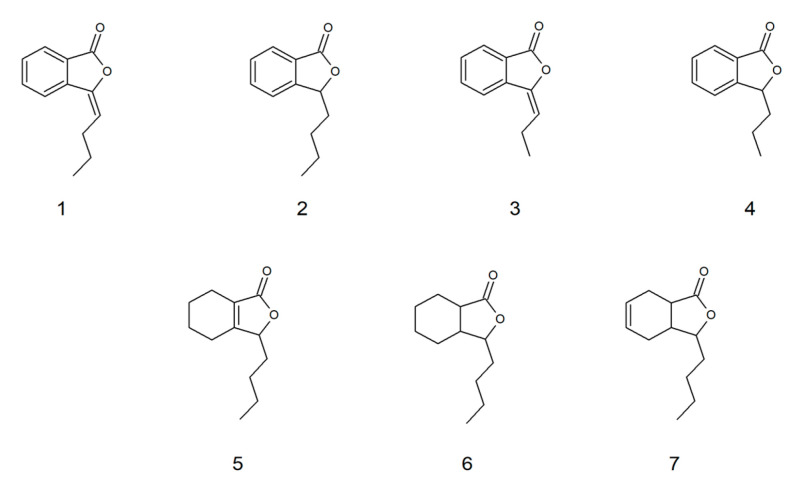
Structures of lactones **1**–**7** used to test fungistatic activity.

**Figure 4:**
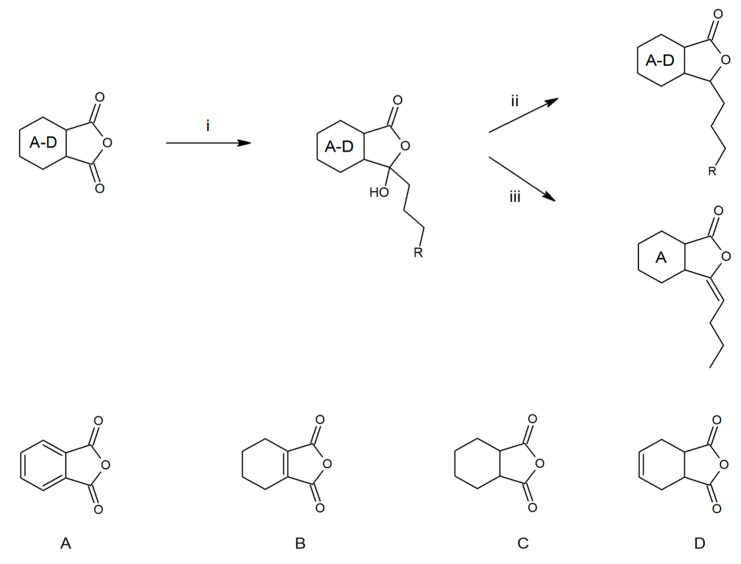
Synthetic route for the obtainment of phthalide lactones. R = H or Me; i-Et_2_O, Mg, *n*-C_4_H_9_Br or C_3_H_7_Br, CdCl_2_; ii-THF, NaBH_4_; iii-toluene, TsOH.

The following compounds were used to test fungistatic activity: 3-*n*-butylidenephthalide (**1**), 3-*n*-butylphthalide (**2**), 3-*n*-propylidenephthalide (**3**), 3-*n*-propylphthalide (**4**), 3-*n*-butyl-4,5,6,7-tetrahydrophthalide (**5**), 3-*n*-butyl-hexahydrophthalide (**6**), and 3-*n*-butyl-3a,4,7,7a-tetrahydrophthalide (**7**) (Figure 3). Lactones, except lactone **3**, which was purchased, were obtained in two-stage syntheses with a yield of 40–60%, in which the intermediate product 3-butyl-3-hydroxylactone or 3-propyl-3-hydroxylactone formed from dialkylcadmium and the appropriate anhydride was subjected to reduction with NaBH_4_ or dehydration with *p*-toluenesulfonic acid (Figure 4) [16]. The structures of the compounds were confirmed using ^1^H NMR, ^13^C NMR, and HR-ESI-MS/MS analyses performed using an ESI-Q-TOF mass spectrometer.

### 2.2. Fungistatic Activity against R. mucilaginosa IHEM 18459 and Correlation with Structure and In Silico Data

IC_50_ values for phthalide lactones **1**–**7** and fluconazole (**8**) against *R. mucilaginosa* IHEM 18459 were determined using the broth microdilution method (Table 1). As *Rhodotorula* spp. may grow in a broad range of temperatures from 5 to 35 °C, and the optimal value is species-dependent [19], microdilution tests were conducted in temperatures of both 25 °C and 30 °C.

Six of the seven lactones inhibited the mycelial growth of *R. mucilaginosa* IHEM 18459 at concentrations ranging from 13 to 66 µg/mL. In contrast, fluconazole (**8**), most commonly used for yeast infections, reduced mycelial growth at a concentration of 199–207 µg/mL.

Data on absorption, distribution, metabolism, and excretion (ADME) parameters are crucial for drug development [20]. Therefore, in silico ADME analysis was included in the study (Table 1). Among these parameters, lipophilicity, solubility, toxicity to rat cells, and inhibition of isoenzymes of P450 cytochrome, which are responsible for the biotransformation and detoxification of drugs, are frequently tested [20,21,22,23].

In addition to the analysis of the differences in the structure of compounds, we analyzed the influence of structure on biological activity. Lactones **1**–**4** are flat aromatic compounds, and cis lactones **5**–**7** with a cyclohexane or cyclohexene ring form conformations intermediate between the chair and boat forms, depending on the environment. With such minor differences in biological activity, it should be noted that lactones **1** and **3**, with an aromatic ring and a double bond at the three-carbon atom, were the most active and three times less toxic than fluconazole (**8**). In contrast, lactones **2** and **4**–**7** were seven to nine times less toxic than fluconazole (**8**). Considering the experimental data, the studies on the toxicity of 3-*n*-butylphthalide (**2**) were conducted by Xue et al., 2011—the IC_50_ values for both primary rat and human hepatocytes were described as above 500 µM, which was the highest concentration tested. This phthalide has been accepted as the treatment of the brain ischemia after clinical studies [24,25]. 

Aromatic lactones **1**–**4** inhibited the enzyme CYP1A2, which means that they might prolong the metabolism of some classes of drugs, including analgesic, antipsychotic, anti-Parkinson, anti-Alzheimer, and anticancer drugs. In contrast, CYP1A2 inhibitors prevent the bioactivation of procarcinogens [26]. Lactones **2**, **6**, and **7** reduced the activity of CYP2C9 and therefore may influence the metabolism of proton pump inhibitors, antiepileptic agents, and antiplatelet agents [27]. In contrast to fluconazole (**8**), the lactones did not reduce CYP2C19 activity. CYP2D6 and CYP3A4 were not inhibited by any of the compounds tested. 

The lowest fungistatic activity was observed for 3-n-propylphthalide (**4**). This compound has a low lipophilicity (LogP = 2.44) compared to the rest of the lactones (log P = 2.78–2.96), which is less soluble in fat than other lactones. All lactones **1**–**7** are poorly soluble in water, although lactones **3**–**4** with a three-carbon side chain differ slightly in this matter.

### 2.3. Determination of Fungistatic Activity for 3-n-Butylidenephthalide *(****1****)* against R. mucilaginosa IHEM 18459 In Vitro and in Food Matrices

The IC_50_ values of compounds **1**–**7** against *R. mucilaginosa* IHEM 18459 were determined using the broth microdilution method. The main advantages of this method include the low amount of compounds required for testing and the low cost; nevertheless, some drawbacks related to inoculum viability and solvent-inhibiting properties occur [28]. Moreover, it has been stated that the incubation temperature, inoculum size, and culture medium may influence the inhibitory concentrations using the microdilution method [29,30].

In connection with the discussion on the advantages and limitations of the broth microdilution method for the most active compound, 3-*n*-butylidenephthalide (**1**), we determined the fungistatic activity based on the weight of the mycelia cultured in various lactone **1** concentrations. Moreover, we discussed the contribution of phthalide **1** to the inhibition of *R. mucilaginosa* using the plate count method and determined yeast growth in dairy food matrices.

#### 2.3.1. Determination of Dry Yeast Biomass

To evaluate the effect of the addition of different concentrations of 3-*n*-butylidenephthalide (**1**) on the dry yeast biomass, we performed two experiments in 0.3 and 2 L flasks with 50 mL and 500 mL Sabouraud medium with inoculum 0.5% vol. and 10% vol., respectively. 

**Figure 5:**
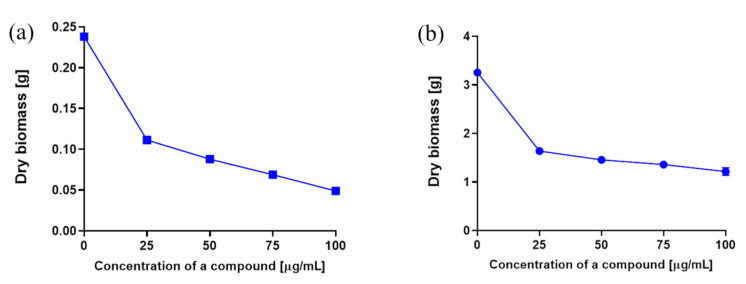
Biomass weight depending on the concentration of compound **1** used, cultivation of *R. mucilaginosa* using different inoculum (% volume medium): (**a**) 0.5% and (**b**) 10%.

The total dry biomass of the control samples were 0.238 and 3.254 g, respectively (Figure 5). In both experiments, a marked decrease in yeast biomass by almost half of the control value was noted for the lowest concentration of 3-*n*-butylidenephthalide (**1**)—25 µg/mL. The use of higher concentrations resulted in subsequent but smaller decreases in dry matter values. Fluconazole (**8**) at a concentration of 50 µg/mL caused only a slight inhibition compared with the control—the amount of reduction of dry biomass was 0.031 and 0.025 g, depending on the inoculum volume (0.5% and 10% volume of the medium, correspondingly).

#### 2.3.2. Plate Count Method In Vitro and in Food Matrices

**Figure 6:**
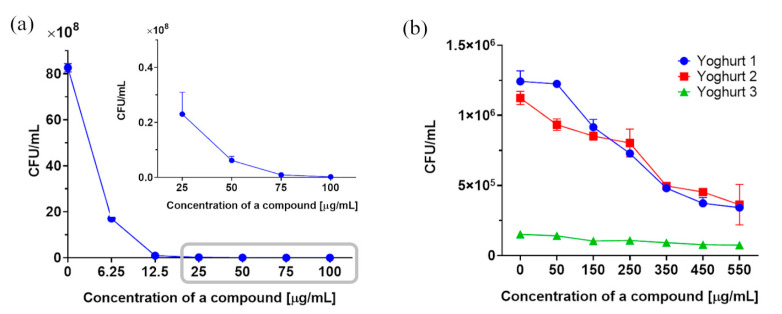
Total yeast cell counts (CFU/mL) depending on the concentration of compound **1** using plate count method (**a**) in vitro and (**b**) in three types of yogurts. The corresponding data represent the mean  ±  standard deviation.

The total number of yeasts (CFU/mL) depending on the concentration of compound **1** used for the two assays is shown in Figure 6.

The number of colonies in the control sample was 8.26 × 10^9^ CFU/mL. We showed that 3-*n*-butylidenephthalide (**1**) caused a notable reduction in this value at the lowest tested concentration of 6.25 µg/mL to the amount of 1.70 × 10^9^ CFU/mL, which means that the 50% inhibition of CFU/mL is below this dose. Fluconazole (**8**), tested at a dose of 50 µg/mL, reduced the number of colonies up to 5.87 × 10^9^ CFU/mL, i.e., by 20% (Figure 6a).

The antimicrobial activity of 3-*n*-butylidenephthalide (**1**) against *R. mucilaginosa* IHEM 18459 was evaluated in food matrices (Figure 6b). Following the promising results of 3-*n*-butylidenephthalide (**1**) in vitro tests, we decided to evaluate the functional antifungal properties of three commercially available yogurt types (1 and 2 are thick yogurts and 3 is a drinking yogurt). Because dairy-derived *Lactobacillus* spp. can affect yeast growth, the results were compared with those of the control [31]. No inhibition was observed using compound **1** at concentrations below 50 µg/mL in preliminary tests. Therefore, phthalide was tested at higher concentrations of 50–550 µg/mL.

The food matrices allowed *Rhodotorula* to grow from 1.12 × 10^6^ CFU/mL for thick yogurt 2 to 1.51 × 10^5^ CFU/mL for drinking yogurt 3. Lactone **1** at concentrations above 50 µg/mL caused a considerable decrease in yeast colonies compared with the control. Inhibition of CFU/mL by half occurred at a concentration of 220–250 µg/mL (1.2–1.3 mM) for thick yogurt. Lactone may serve as a promising alternative to sodium chloride, potassium sorbate, sodium acetate, sodium benzoate, and other preservatives used in dairy products [32].

The differences in activity between the in vitro assay and the assay that mimics the target conditions may be due to the uneven distribution of the antifungal agent in the food matrix. This corresponds to the relatively high lipophilicity of 3-*n*-butylidenephthalide (**1**) associated with its poor solubility in water [33]. Blanco-Padilla pointed out that a potential problem with natural antimicrobials could also be their stability and drew attention to nanocarriers to improve cell delivery [34]. However, the amount of 3-*n*-butylidenophthalide (**1**) used is still low, and considering its low toxicity, it can be recommended for use.

### 2.4. Microbial Transformation 3-n-Butylidenephthalide *(****1****)* by R. mucilaginosa 

We examined whether the yeast *R. mucilaginosa* IHEM 18459 transformed 3-*n*-butylidenephthalide (**1**). Biotransformations were carried out for eight days. The cultures to which lactone **1** was added were centrifuged and separated from the mycelium and supernatant using ethyl acetate. We found that 90% of the lactone was accumulated in the yeast mycelium. A slight loss of substrate over time was observed. The above results indirectly indicate that 3-*n*-butylidenephthalide (**1**) with log P 2.93 and log S −3.3, which is highly soluble in lipids, was accumulated in the cytosol.

### 2.5. The Influence of the 3-n-Butylidenephthalide *(****1****)* on ROS Level

**Figure 7:**
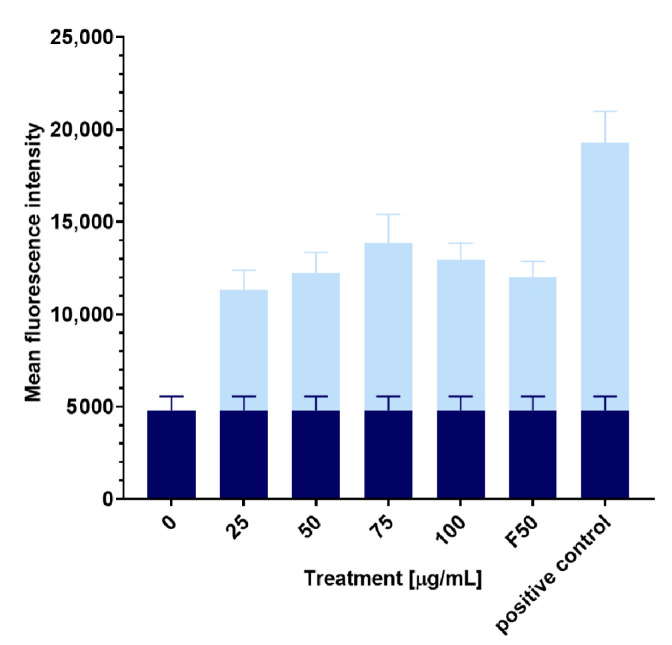
Reactive oxygen species (ROS) levels with different concentrations of added compounds. Control levels of ROS are marked by the dark blue color. F means fluconazole. Data represent the mean  ±  standard deviation.

Physiological ROS are necessary for proper cell functioning, including signaling pathways [35]. However, according to the research conducted by Gong et al., 3-*n*-butylphthalide (**2**) antifungal activity is likely associated with intracellular ROS overproduction, which leads to mitochondrial dysfunction [13]. The damaging effects of ROS inside cells and mitochondria include structural changes in DNA and RNA, peroxidation of membrane phospholipids (consisting of polyunsaturated fatty acids), and oxidation of cellular proteins that perform numerous functions inside the cell [36]. ROS values result from the xenobiotic effect and the well-known antioxidative properties exhibited by carotenoids, including α- and *β*-carotene and lycopene [37]. It has been stated that supplementation with some antioxidants, e.g., ascorbic acid, may reduce fluconazole-induced inhibition [38]. The 4 h treatment of *R. mucilaginosa* IHEM 18459 cells with 3-*n*-butylidenephthalide (**1**) or fluconazole (**8**) increased the level of intracellular ROS up to 53% compared with the control at a concentration of 75 µg/mL (Figure 7). Fluconazole (**8**) at 50 µg/mL also caused elevated levels of oxidative stress. An increase in ROS under the influence of fluconazole was observed in experiments using *Candida tropicalis*, *C. albicans*, and *Cryptococcus neoformans* [39].

**Figure 8:**
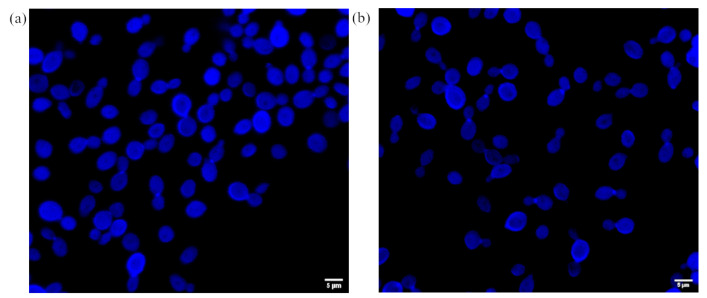
Calcofluor staining of the *R. mucilaginosa* cells: (**a**) the control, and (**b**) sample with the addition of 100 µg/mL 3-*n*-butylidenephthalide (**1**).

We evaluated whether the effect of 3-*n*-butylidenephthalide (**1**) on yeast cells can be visualized using confocal microscopy with Calcofluor White staining. This dye was used previously by Dagher et al. [40] and Suchodolski et al. [41] for staining yeast cell walls as it binds with chitin. However, we did not observe a change in the fluorescence intensity between the control and tested samples (Figure 8).

### 2.6. Synergistic Effect of 3-n-Butylidenephthalide *(**1**)* and Fluconazole *(**8**)*

The checkerboard protocol previously described in the literature was used to determine the effects of 3-*n*-butylidenephthalide (**1**) and fluconazole (**8**) in combination [15,42].

**Figure 9:**
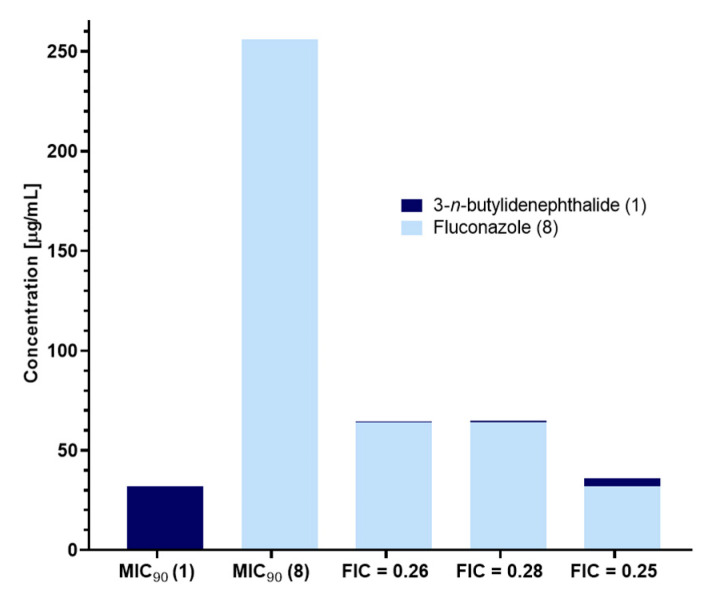
Synergistic effect of 3-*n*-butylidenephthalide (**1**) and fluconazole (**8**); MIC_90_—the lowest concentration of agent at which 90% of the yeasts were inhibited; and FIC—fractional inhibitory concentration.

Fractional inhibitory concentration values less than 0.5 indicated that phthalide **1** and fluconazole (**8**) showed a synergistic effect. The compounds inhibited the growth of *R. mucilaginosa* IHEM 18459 by 90% when tested in combination at lower concentrations, e.g., 4 µg/mL of lactone **1** and 32 µg/mL of fluconazole (**8**), instead of doses of 32 µg/mL and 256 µg/mL for the compounds administered separately (Figure 9). This action greatly reduces drugs from the azole group, causing drug resistance [13,15].

### 2.7. The Influence of the 3-n-Butylidenephthalide *(**1**)* on the Production of Carotenoids

*R. mucilaginosa* is known to produce carotenoids [43]. As we observed changes in the color of the cultures depending on the compound **1** concentration used, we investigated the *β*-carotene content and overall carotenoid composition in those samples. The connection between carotenoid profiles and factors triggering oxidative stress has been reported in the literature [44].

Kot et al. noted that the pigments are deposited in lipid bodies; therefore, an appropriate cell disruption method is required before extraction [45]. Indeed, direct extraction of biomass using dimethyl sulfoxide with incubation at 50 °C and subsequent extraction using acetone was not sufficient, as some pigments remained in the cells. Therefore, we implemented ultrasonic disintegration of yeast cells, followed by centrifugation and vortexing with dimethyl sulfoxide (DMSO) and Folch solution until the biomass was colorless. The resulting solution was evaporated, dissolved in acetonitrile, and subjected to APCI-LC-HR-MS. The analysis was targeted to detect the selected pigments present in the *Rhodotorula* pathway, according to Kot et al. [45] and Moliné et al. [46]. Briefly, colorless phytoene, the first 40-carbon compound of this route, is converted to neurosporene by desaturase. Subsequently, transformations to lycopene or, on some occasions, *β*-zeacarotene occur, followed by a series of cyclization reactions to *γ*- and *β*-carotene. In parallel, β-carotene is converted to torulene, from which derivatives are formed by hydroxylation and oxidation, including torularhodin [45,46].

**Figure 10:**
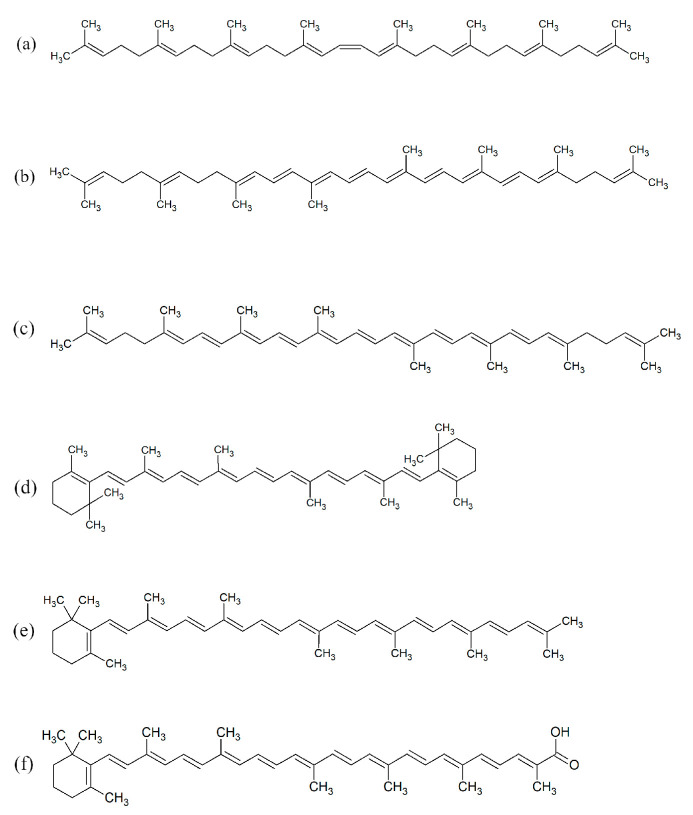
The structures of carotenoids detected in *R. mucilaginosa* IHEM 18459 biomass: (**a**) phytoene, (**b**) neurosporene, (**c**) lycopene, (**d**) *β*-carotene, (**e**) torulene, and (**f**) torularhodin.

The APCI-LC-HR-MS results of the control sample indicated the presence of the precursors phytoene, neurosporene, lycopene, *β*-carotene, torulene, and torularhodin (Figure 10). β-zeacarotene was not detected. *β*-carotene and lycopene were additionally confirmed using high-performance liquid chromatography (HPLC)-DAD based on a comparison with the retention time of the compound standards.

The differences in particular pigments between the samples were visualized using quantitative comparative analysis (Table 2). The relative quantity of the compounds was defined as 1 for the control sample, and the remaining samples were multiples of this number within each row.

Our results confirmed a change in pigment formation in cultures supplemented with 3-*n*-butylidenephthalide (**1**). Similarly, Moț et al. showed that antifungal naftifine caused dose-dependent depigmentation of cells. A possible mechanism of action involves compound-induced ROS formation, triggering the carotenoid defense response [47]. Another known inhibitor of carotenogenesis is diphenylamine, which inhibits the desaturation processes affecting phytoene synthase, leading to the accumulation of phytoene [46]. Additionally, the content of the carotenoid precursor in samples with the addition of 3-*n*-butylidenephthalide (**1**) increased up to 96 times when the inhibitor was tested at a concentration of 75 µg/mL. This result may indicate the same mechanism of action as for diphenylamine. The next carotenoid in the pathway, neurosporene, was detected in only one sample, in which the highest content of its precursor was also detected. The production of *β*-carotene and lycopene was over fourfold increased when a lower concentration of compound **1** was applied; however, it decreased along with the addition of a higher concentration of the inhibitor, leading to even five times less *β*-carotene production than the control sample. Torulene and torularhodin were formed in reduced quantities at all the 3-*n*-butylidenephthalide (**1**) concentrations tested. At 100 µg/mL, only trace amounts of torulene were noted, whereas torularhodin was not detected.

According to the literature, ketoconazole at doses of 20–50 mg/L significantly increased the content of pigments, interpreted as overexpression of the HMG1 gene increasing the activity of HMG-CoA reductase, which results in enhancement of the pigment precursor [48]. We observed that the addition of fluconazole (**8**) also led to increased amounts of colored *β*-carotene and lycopene, torulene, and torularhodin by up to 2.30, 1.60, and 1.63 times, respectively, as compared with the control. Fluconazole (**8**) did not induce phytoene accumulation to the same extent as phthalide **1**, causing only a threefold increase.

By analyzing the data on *β*-carotene content in the samples determined using HPLC-DAD, it can be concluded that, similar to the previous experiment, the smallest dose of 3-*n*-butylidenephthalide (**1**) caused an increase in *β*-carotene content in comparison with the control (Table 3). In addition, fluconazole (**8**) greatly increased pigment production. This enhancement of *β*-carotene production using a medium containing compound **8** is consistent with the findings of Li et al. [49]. Interestingly, the application of phthalide **1** at a higher concentration of 75 µg/mL caused a twofold decline, whereas, at a dose of 100 µg/mL, carotenoids were not detected using the HPLC-DAD method.

### 2.8. The Influence of the 3-n-Butylidenephthalide *(**1**)* on the Production of FAME

Lipid biosynthesis occurs in the cytoplasm of yeast cells through enzyme-derived sequences that form biosynthetic substrates of saccharides, glycerol, or acetyl-CoA to long-chain fatty acids. This process is closely related to glycolysis and the Krebs cycle [50,51].

*R. mucilaginosa* and other members of this genus are considered oleaginous yeasts [52]. Lipids, with special emphasis on sterols, amphipathic phospholipids, and fatty acids (FAs) as their components, are essential for membrane creation [53]. *Rhodotorula* strains store up to 40% of lipids in dry matter, of which triglycerides constitute 65–90% of their content [2]. 

The efficiency and effectiveness of lipid biosynthesis in *R. mucilaginosa* are determined by several factors. First, a high molar C/N ratio in the culture medium is a key factor in the development of lipid-accumulating yeasts. *R. mucilaginosa* LP-2 has been observed to produce 46.7% lipid levels at a C/N ratio of 65 and 35.7% at a ratio of 25 [54]. In this study, we used a C/N ratio of 23, which was constant in all experiments. In addition, carbon sources play an important role [55]. The third important parameter influencing the fatty acid profile was the yeast incubation temperature. In Adel et al. [56], a study conducted at three temperatures of 7 °C, 15 °C, and 26 °C, it was shown that the most unsaturated acids 63.4% are obtained at 15 °C, compared with 57.4% at 7 °C and 43.5% at 26 °C. All our experiments regarding FAME analyses were carried out at the temperature of 25 °C.

**Figure 11:**
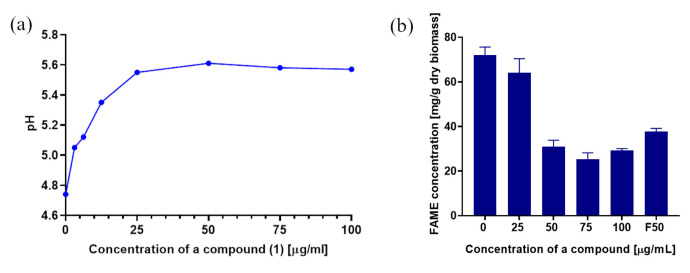
(**a**) The change in pH values of *R. mucilaginosa* cultures with different concentrations of 3-*n*-butylidenephthalide (**1**), and (**b**) FAME content depending on different doses of the compounds (F means fluconazole). The data are shown as the mean ± standard deviation.

The key parameter that affects lipid biosynthesis is pH. Karatay and Donmez [57] conducted various experiments at pH values ranging from 4 to 7. The maximum content of lipids in *Rhodotorula* biomass was obtained at pH = 5, equaling 69.5%, while the pH 4, 6, and 7 resulted in the obtainment of 50.8%, 51.7%, and 64.5%, respectively. In this study, the pH of the control sample was 4.75 and changed with increasing concentrations of 3-*n*-butylidenephthalide (**1**) up to 25 µg/mL. In the concentration range of 25–100 µg/mL, the pH remained relatively stable near the value of 5.5 ± 0.1 (Figure 11a).

Another crucial factor is the increase in ROS inside the cell and mitochondria under the influence of 3-*n*-butylidenephthalide (**1**). ROS are responsible for lipid peroxidation, changing the quantitative and qualitative composition and contributing to the disintegration of cell membranes [36].

The addition of azoles may cause a shift in the desaturation and elongation of FAs, impacting membrane function and growth [58]. To determine the concentration and change in the FA profile between the tested samples, the dry biomass of *R. mucilaginosa* was subjected to alkaline hydrolysis and methylation to obtain FAMEs. The use of glucose-rich Sabouraud medium resulted in a total FAME yield of 75.65 mg/g dry biomass in the control sample (Figure 11b). Khot [52] reported maximum FAMEs content in *R. mucilaginosa* IIPL32 at 97.23 mg/g dry biomass; however, it has been stated that lipid composition varies depending on aeration, medium composition, and incubation time [59]. During the experiment, no considerable difference was noted for 3-*n*-butylidenophthalide (**1**) dosed at 25 μg/mL in the medium as compared with the control. Nevertheless, there was a decline in FAME content by nearly 57% with compound **1** addition in the concentration of 50 μg/mL. The reference antimycotic fluconazole (**8**) at this dose caused a smaller decrease. The concentrations of 75 and 100 μg/mL of 3-*n*-butylidenophthalide (**1**) caused a similar effect in FAME reduction, up to 66% in relation to the control (Figure 11b).

**Figure 12:**
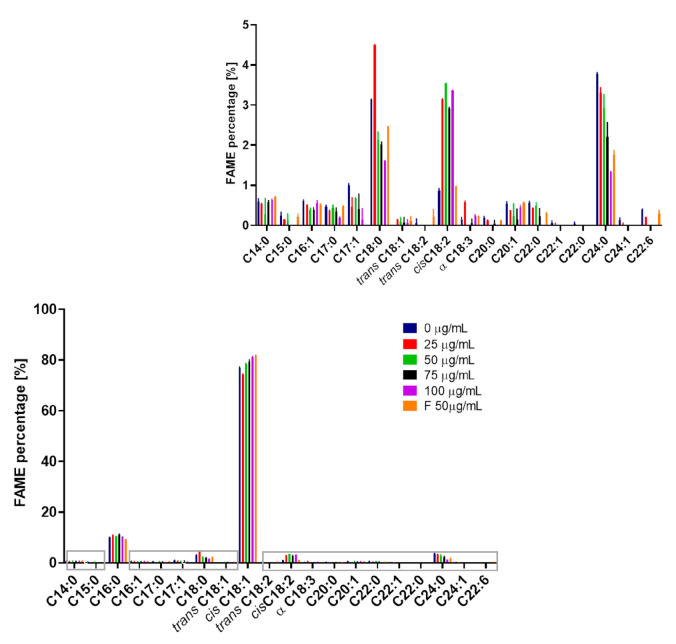
FAME percentage depending on the addition of compounds to the culture (F means fluconazole).

The vast majority (79.49%) of the control sample consisted of monounsaturated fatty acid esters, while the polyunsaturated accounted only for 1.49%. The predominant FAME detected included oleic acid (*cis* C18:1) ester with a concentration of 77.15; next, in descending order of proportion, were palmitic (C16:0), lignocerate (C24:0), stearic (C18:0), and linoleic (C18:2 *n*-6) methyl esters (Figure 12).

Other detected FAMEs did not exceed 1% separately. Among these, only negligible amounts of the odd-chain fatty acids C15:0, C17:0, and C17:1 were formed. Their microbial production is very limited; nevertheless, attempts to increase their yield have been made through propionate addition or genetic engineering [60]. 

Similarly, Ayadi et al. [61] noted that *cis* C18:1 and C16:0 were dominant in *R. mucilaginosa* Y-MG1 biomass with 58.30% and 15.20%, respectively, of overall FAMEs content, using a synthetic medium with glucose as the carbon source. Other FAMEs previously reported in the literature in various *R. mucilaginosa* strains included palmitoleic (C16:1), linoleic (C18:2), and other C10-18 esters present in a minority [52,59,61].

As the distribution of saturated and unsaturated lipids is crucial for membrane fluidity, we examined the proportions of these components [62]. Considering the impact of the dose of the compounds on the profile of FAMEs, the control sample contained 19.02% of saturated FAs, but the highest percentage of saturated fatty acids (20.31%) was recorded for the addition of compound **1** at a concentration of 25 µg/mL. The proportion further decreased with increasing levels of 3-*n*-butylidenephthalide (**1**), except 75 µg/mL, which showed no considerable difference compared with 50 µg/mL. A reduction in the saturated FAs content was noted at a dose of 100 µg/mL, resulting in a content of 14.12%. The addition of fluconazole (**8**) also notably declined their share to 15.27%. As unsaturation increased, the proportion of polyunsaturated FAs increased when using compound **1** compared with the control, although there was no straightforward dose dependency. Fluconazole (**8**) did not cause notable shifts in the polyunsaturated FAs content between the control and tested groups (Table 4).

The rising proportion of unsaturated FAs suggests an increase in membrane fluidity, as phospholipids with these FAs have relatively lower melting points [63]. An explanation for the increased share of unsaturated FAs with increasing concentration of added compound **1** may be the influence on the expression of genes encoding fatty acid desaturases. These enzymes catalyze the formation of unsaturated acids from their saturated analogues, and their expression in the OLE pathway may be affected by nutrient and environmental factors [64]. For instance, Δ9-fatty acid desaturase is responsible for the formation of palmitoleic acid (C16:1) and oleic acid (*cis* C18:1) [65]. The increase in the percentage of *cis* C18:1 acid with the simultaneous decline of saturated analogue C18:0 (Figure 12) suggests the involvement of 3-*n*-butylidenephthalide (**1**) in the OLE pathway.

### 2.9. The Influence of the 3-n-Butylidenephthalide *(**1**)* on the Ergosterol Content

The main antifungal mechanism of action of azoles includes blocking the sterol pathway by lowering lanosterol 14*α*-demethylase activity (Erg11p). Consequently, another toxic sterol is created by other enzymes in this pathway, which leads to the inhibition of cell growth or death [66,67].

**Figure 13:**
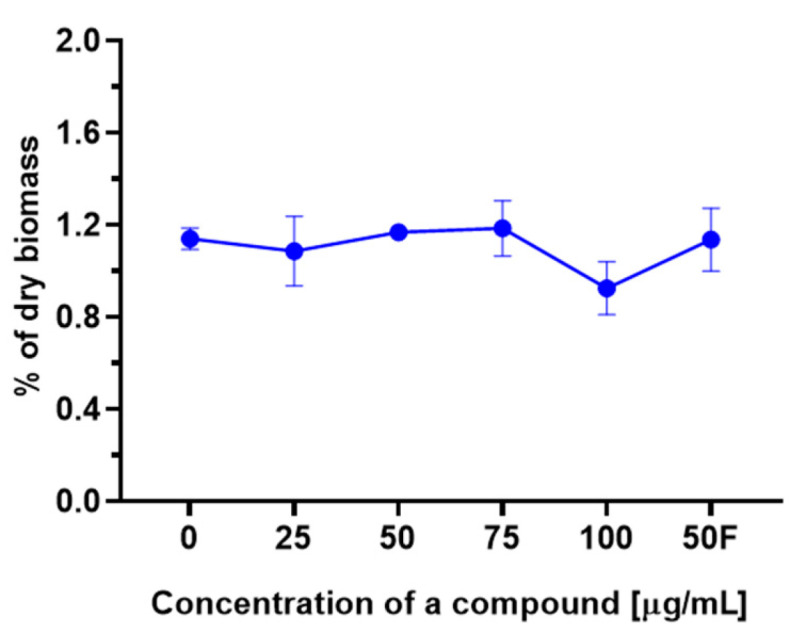
Ergosterol content [% of dry biomass] depending on the concentration of the added compound. The data are shown as the mean ± standard deviation.

However, 3-*n*-butylidenephthalide (**1**) did not notably change ergosterol content (Figure 13). This indicates that the observed fungistatic activity of lactones **1**–**7** is not related to the disintegration of the cell membrane associated with the lack of ergosterol.

## 3. Materials and Methods

### 3.1. General

Solvents used in this study were purchased from Merck (Darmstadt, Germany). 3-*n*-Propylidenephthalide (**3**) was purchased from Sigma-Aldrich (St. Louis, MO, USA). Fluconazole (**8**) was purchased from TCI (Tokyo, Japan). Supelco 37 Component FAME Mix, *β*-carotene (European Pharmacopoeia (EP) Reference Standard), Calcofluor White, 2ʹ,7ʹ-dichlorofluorescin diacetate (H_2_DCFDA), phosphate-buffered saline (PBS) pH 7.2, and boron trifluoride in diethyl ether (48%) were purchased from Merck (Darmstadt, Germany). Preparative column chromatography of the lactones after synthesis was performed using Silica Gel (Kieselgel 60, 230–400 mesh, 40–63 µm, Merck).

The following microbiological media were used: RPMI 1640 medium buffered with 3-(N-morpholino)propanesulfonic acid (MOPS): 10.4 g RPMI 1640 with l-glutamine, without sodium bicarbonate (Sigma-Aldrich, St. Louis, MO, USA), 34.53 g MOPS (Pol-Aura, Dywity, Poland), 1 L distilled water; pH adjusted to 7.0, medium was sterile-filtered; Sabouraud medium: 30 g glucose (Chempur, Piekary Śląskie, Poland), 10 g peptone and 2 g bacteriological LAB-AGAR™ (Biomaxima, Lublin, Poland), and 1 L of distilled water; pH was adjusted to 5.6.

The strain used for the study was *R. mucilaginosa* IHEM 18459 derived from Belgian Coordinated Collections of Microorganisms (BCCM).

Centrifugation of samples was carried out using an Eppendorf centrifuge 5810 R (Hamburg, Germany). The yeast biomass was lyophilized using a LYO SRK GT2 Basic (Riedstadt, Germany). Incubation of microtiter plates for the broth microdilution test took place in a Biosan PST-60 HL ThermoShaker (Riga, Latvia), while their analysis was performed using a BioTek TS-800 microplate reader (Riga, Latvia). Carotenoids were extracted from the biomass using a Vortex Mixer (VELP SCIENTIFICA, Usmate Velate, Italy). Fluorometric measure for the ROS determination was taken using a Synergy H1 microplate reader (BioTek Instruments, Winooski, VT, USA).

Gas chromatography analyses (GC, FID, carrier gas H_2_) were performed on an Agilent Technologies 8860 (GC System, Santa Clara, CA, USA). For the FAME determination, column Zebron ZB-FAME, 60 m × 0.25 mm × 0.2 µm (Phenomenex, Torrance, CA, USA), while for tests of the purity of compounds **1**–**7**, HP-5 column, 30 m × 0.32 mm × 0.25 μm (Agilent, Santa Clara, CA, USA) were used.

*β*-carotene content and microbial transformations of 3-*n*-butylidenephthalide (**1**) were analyzed using a Dionex UltiMate 3000 instrument with a diode array detector (Thermo Fisher Scientfic, Waltham, MA, USA) with column Agilent Zorbax Bonus-RP 3.5 µm 150 × 3 mm.

HR-APCI–MS/MS analyses of the carotenoids were performed with RSLC UltiMate 3000 (Dionex, Sunnyvale, CA, USA) coupled with mass spectrometer APCI-Q-TOF, maXis impact (Bruker, Billerica, MA, USA). Luna Omega 1.6 µm C18, 50 mm × 2.1 (Phenomenex, Torrance, CA, USA) was the column used for the pigments determination and quantitative comparative analysis. 

The staining of the yeast cells was performed in Millicell^®^ EZ SLIDE (Merck, Darmstadt, Germany), assembled with the microscope Epredia X1000 Coverslip 24 × 50 mm #1.5 (0.16–0.19 mm) slides (Epredia, Kalamazoo, MI, USA). The confocal microscopy imaging was performed using a STEDyCON superconfocal microscope (Abberrior Intruments, Göttingen, Germany) installed on a Nikon Ti2E microscope body with a 100× objective lens (Nikon, Tokyo, Japan).

A Cintra 101 spectrophotometer (GBC Scientific, Braeside, Australia) was used to analyze the ergosterol content of the samples.

^1^H NMR and ^13^C NMR spectra were recorded in CDCl_3_ on a Bruker Avance 500 (500 MHz, Billerica, MA, USA) spectrometer.

### 3.2. Synthesis of Phthalide Lactones

Compounds **1**–**2** and **4**–**7** were synthesized using the method described below:

Step 1: We used the method we described in a previous article [68]. Briefly, synthesis was carried out in a multinecked flask equipped with a dropping funnel, magnetic stirrer core, and reflux condenser. Diethyl ether or THF, freshly distilled from LiAlH_4_, was used as the solvent. Magnesium chips (0.05 M) were activated by heating with a few iodine crystals, and 0.055 mM of the appropriate alkyl bromide was added dropwise. Anhydrous cadmium chloride (0.025 M) was added to the obtained Grignard compound, and the mixture was heated for 1 h. After cooling, 0.05 M of an appropriate anhydride was added dropwise. The reaction mixture was heated for 6 h. A 10% HCl solution was added to the cooled reaction mixture for acidification. The solvents were evaporated on a rotary evaporator, and the reaction residue was extracted using a separatory funnel with methylene chloride. The collected organic layer was dried over anhydrous MgSO_4_, filtered through a filter paper, and evaporated.

Step 2a (compounds **2**; **4**–**7**): The concentrated mixture was dissolved in 100 mL of THF, followed by the addition of 6 mM NaBH_4_. The mixture was stirred under reflux for 8 h. The mixture was acidified with 10% HCl and stirring was continued. The solvent was then evaporated and the residue was extracted using a separatory funnel with methylene chloride. The residue was purified using liquid column chromatography.

Step 2b (compound **1**): The mixture was dissolved in 300 mL of toluene in a round-bottomed flask equipped with an azeotropic cap, after which 1 mM *p*-toluenesulfonic acid was added and heated with a heating mantle until the substrate has completely reacted, that is, until the water stopped evaporating. 

The obtained compounds were purified via liquid column chromatography using hexane/isopropanol/acetone/ethyl acetate (60:3:1:1) as the eluent.

For the HR-ESI–MS/MS analyses of lactones **1**–**7**, the major operating parameters were as follows: flow rate of the sample: 180 µL/min; nebulizer pressure: 0.4 bar, heating gas flow: 3.0 L/min, heating gas temperature: 180/200 °C; data acquisition range: *m*/*z* 50–1300/1400 *m*/*z*; ionization mode: positive and negative; and ion source energy: 5 eV. 

The additional spectral data are presented in the attached Supplementary Materials.

3-*n*-butylidenephthalide (**1**) was prepared according to the procedure (steps 1 and 2b), and C_4_H_9_Br and phthalic anhydride (7.5 g, 0.05 mole) were used as the substrate. An amount of 3.57 g (yield 38%) of phthalide was obtained as a mixture of two diastereoisomers. Bioactivity of compound **1** was tested for the mixture of isomers *E* and *Z*, of percentage composition 8 and 92%, correspondingly.

Spectroscopic data: ^1^H NMR (500 MHz, CDCl_3_), δ (ppm): 0.98 (t, 3, *J* = 7.4, CH_3_-11), 1.54 (m, 2, CH_2_-10), 2.45 (q, 2, *J* = 15.0, 7.5, CH_2_-9), 5.63 (t, 1, *J* = 7.8, CH-8), 7.49 (t, 1, *J* = 7.3, CH-5), 7.62–7.69 (m, 2, CH-4), 7.62–7.69 (m, 2, CH-6), 7.88 (d, 1, *J* = 7.7, CH-7). ^13^C NMR (151 MHz), δ (ppm): 13.9 (C-11), 22.6 (C-10), 28.0 (C-9), 109.6 (CH-8), 119.8 (CH-4), 124.6 (C-7a), 125.4 (C-7), 129.5 (CH-6), 134.3 (CH-5), 139.7 (C-3a), 145.9 (C-3), 167.3 (C-1). HRMS (*m*/*z*): [M + H]^+^ 189.0908 (experimental), 189.0910 (calculated); formula: C_12_H_12_O_2_.

3-*n*-butylphthalide (**2**) was prepared according to the procedure (steps 1 and 2a), and C_4_H_9_Br and phthalic anhydride (7.5 g, 0.05 mole) were used as the substrate. An amount of 4.65 g (yield 48.8%) of phthalide was obtained. Spectroscopic data: _1_H NMR (500 MHz, CDCl_3_), δ (ppm): 0.91 (t, 3, *J* = 7.2, CH_3_-11), 1.31–1.44 (m, 2, CH_2_-10), 1.44–1.54 (m, 2, CH_2_-9), 1.65–1.80 (m, 1, one of CH_2_-8), 1.99–2.12 (m, 1, one of CH_2_-8), 5.47 (dd, 1, *J* = 7.9, 3.7, CH-3), 7.44 (d, 1, *J* = 7.7, CH-4), 7.52 (t, 1, *J* = 7.5, CH-6), 7.66 (t, 1, *J* = 7.5, CH-5), 7.89 (d, 1, *J* = 7.7, CH-7). ^13^C NMR (151 MHz), δ (ppm): 14.0 (C-11), 22.6 (C-10), 27.0 (C-9), 34.6 (C-8), 81.6 (CH-3), 121.8 (CH-4), 125.8 (CH-7), 126.3 (C-7a) 129.1 (CH-6), 134.1 (CH-5), 150.3 (C–3a), 170.8 (C-1). HRMS (*m*/*z*): [M + H]^+^ 191.1065 (exp.), 191.1067 (calc.); formula: C_12_H_14_O_2_.

3-*n*-propylphthalide (**4**) was prepared according to the procedure (steps 1 and 2a), and C_3_H_7_Br and phthalic anhydride (7.5 g, 0.05 mole) were used as the substrate. An amount of 3.61 g (yield 42%) of phthalide was obtained. Spectroscopic data: ^1^H NMR (500 MHz, CDCl_3_), δ (ppm): 0.98 (t, 3, *J* = 7.4, CH_3_-10), 1.46–1.58 (m, 2, CH_2_-9), 1.72–1.78 (m, 1, one of CH_2_-8), 1.98–2.04 (m,1, one of CH_2_-8), 5.48 (dd, 1, *J* = 8.0, 4.0, CH-3), 7.44 (d, 1, *J* = 7.7, CH-4), 7.52 (t, 1, *J* = 7.5, CH-6), 7.66 (t, 1, *J* = 7.5, CH-5), 7.89 (d, 1, *J* = 7.7, CH-7). ^13^C NMR (151 MHz), δ (ppm): 13.97 (CH_3_-10), 18.41 (CH_2_-9), 36.99 (CH_2_-8), 81.42 (CH-3), 121.87 (CH-4), 125.88 (CH-7), 126.32 (C-7a), 129.18 (CH-6), 134.08 (CH-5), 150.31 (C-3a), 170.86 (C-1). HRMS (*m*/*z*): [M + H]^+^ 177.0908 (exp.), 177.0910 (calc.); formula: C_11_H_12_O_2_.

3-*n*-butyl-4,5,6,7-tetrahydrophthalide (**5**) was prepared according to the procedure (steps 1 and 2a), and C_4_H_9_Br and 3,4,5,6-tetrahydrophthalic anhydride (7.7 g, 0.05 mole) were used as the substrate. An amount of 4.37 g (yield 45.1%) of phthalide was obtained. Spectroscopic data: ^1^H NMR (500 MHz, CDCl_3_), δ (ppm): 0.88 (t, 3, *J* = 7.1, CH_3_-11), 1.22–1.40 (m, 4, CH_2_-10, CH_2_-9), 1.41–1.50 (m, 1, one of CH_2_-8), 1.59–1.77 (m, 4, CH_2_-5, CH_2_-6), 1.79–1.87 (m, 1, one of CH_2_-8), 2.13–2.24 (m, 4, CH_2_-4, CH_2_-7), 4.74–4.79 (m, 1, CH-3). ^13^C NMR (151 MHz), δ (ppm): 13.77 (CH_3_-11), 19.77 (CH_2_-7), 21.66 (CH_2_-5), 21.69 (CH_2_-6), 22.52 (CH_2_-10), 23.25 (CH_2_-4), 26.66 (CH-9), 32.08 (CH-8), 83.01 (C-3), 126.49 (C-7a), 163.84 (C-3a), 173.85 (C-1). HRMS (*m*/*z*): [M + H]^+^ 195.1376 (exp.), 195.1380 (calc.); formula: C_12_H_18_O_2._

3-*n*-butyl-hexahydrophthalide (**6**) was prepared according to the procedure (steps 1 and 2a), and C_4_H_9_Br and hexahydrophthalic anhydride (7.8 g, 0.05 mole) were used as the substrates. An amount of 4.70 g (yield 48%) of phthalide was obtained. Spectroscopic data: ^1^H NMR (500 MHz, CDCl_3_) δ (ppm): 0.90 (t, 3, *J* = 7.1 Hz, CH_3_-11), 1.07–1.10 (m, 2, CH_2_-10), 1.29–1.36 (m, 2, CH_2_-7), 1.37–1.65 (m, 8, CH_2_-4, CH_2_-5, CH_2_-6, CH_2_-9), 1.70–1.77 (m, 1, one of CH_2_-8), 2.15–2.18 (m, 1, one of CH_2_-8), 2.29–2.31 (m, 1, CH-3a), 2.72 (t, 1, *J* = 6.2, CH-7a), 4.20–4.26 (m, 1, CH-3). ^13^C NMR (151 MHz) δ (ppm): 13.91. (CH_3_-11), 22.48 (CH_2_-10, CH_2_-9), 22.55 (CH_2_-5), 22.88 (CH_2_-8), 23.58 (CH_2_-6, CH_2_-4), 27.96 (CH_2_-7), 38.95 (CH-3a), 42.08 (CH-7a), 82.03 (C-3), 178.10 (C-1). HRMS (*m*/*z*): [M + Na]^+^ 219.1367 (exp.), 219.1434 (cal.); formula: C_12_H_20_O_2._

3-*n*-butyl-3a,4,7,7a-tetrahydrophthalide (**7**) was prepared according to the procedure (steps 1 and 2a), and C_4_H_9_Br and 1,2,5,6-tetrahydrophthalic anhydride (7.7 g, 0.05 mole) were used as the substrate. An amount of 4.85 g (yield 50%) of phthalide was obtained. Spectroscopic data: ^1^H NMR (500 MHz, CDCl_3_) δ (ppm): 0.91 (t, 3, *J* = 7.1 Hz, CH_3_-11), 1.30–1.40 (m, 3, one of CH_2_-9, CH_2_-10), 1.42–1.50 (m, 1, one of CH_2_-9), 1.52–1.61 (m, 2, one of CH_2_-9, one of CH_2_-8), 1.72–1.79 (m, 1, one of CH_2_-8), 1.80–1.88 (m, 1, one of CH_2_-4), 2.01 (dt, 1, *J* = 17.3, 5.8, one of CH_2_-4), 2.30–2.38 (m, 1, one of CH_2_-7), 2.38–2.46 (m, 1, one of CH_2_-7), 2.49–2.59 (m, 1, CH-3a), 2.79–2.87 (m, 1, CH-7a), 4.32–4.38 (m, 1, CH-3), 5.63–5.75 (m, 2, CH-5, CH-6). ^13^C NMR (151 MHz) δ (ppm): 14.0 (CH_3_-11), 19.72 (CH_2_-10), 22.07 (CH_2_-4), 22.65 (CH_2_-7), 28.09 (CH-7a), 29.00 (CH_2_-9), 35.40 (CH_2_-8), 40.03 (CH-3a), 82.69 (CH-3), 124.45 (CH-5), 125.30 (CH-6), 178.74 (C-1). HRMS (*m*/*z*): [M + Na]^+^ 217.1202 (exp.), 217.1277 (cal.); formula: C_12_H_18_O_2._

### 3.3. Fungistatic Activity against R. mucilaginosa IHEM 18459 and Correlation with Structure and In Silico Data

#### 3.3.1. Broth Microdilution Method

The compounds **1**–**8** were screened for their antimicrobial activity using the microdilution method with modified CLSI guidelines [69]. The broth used for the tests included RPMI 1640 medium buffered with MOPS.

Different concentrations of compounds **1**–**8** were prepared in DMSO and then diluted 50 times in the medium. An amount of 100 microliters of each concentration was placed into the wells of 96-well microplates. *R. mucilaginosa* IHEM 18459 was cultivated on Sabouraud agar. The inoculum was made from a 24 h culture by a dilution to 0.5 McFarland and then further diluted to obtain a density of 1 × 10^3^–5 × 10^3^ CFU/mL. The inoculum volume pipetted into the previously placed antifungals was 100 µL. All the compounds were tested in triplicate. The plates were then incubated separately at 25 °C and 30 °C for 48 h using 1200 RPM. The activity was determined spectrophotometrically at a wavelength of 595 nm and determined as the half-maximal inhibitory concentration (IC_50_).

#### 3.3.2. In Silico ADME Studies

The prediction of lipophilicity (Log P), solubility (log S), and CYP isoenzyme inhibition for compounds **1**–**8** was performed using the tool designed by the Swiss Institute of Bioinformatics at the University of Lausanne (http://www.swissadme.ch/index.php (accessed on 25 February 2023)), reported by Daina et al. [23].

The toxicity of rat cells was tested using the computational tool GUSAR (General Unrestricted Structure-Activity Relationships: http://www.way2drug.com/gusar/index.html (accessed on 25 February 2023)) as part of the Way2Drug platform, as reported by Druzhilovskiy et al. [22].

### 3.4. Determination of Fungistatic Activity for 3-n-Butylidenephthalide *(**1**)* against R. mucilaginosa IHEM 18459 In Vitro and Food Matrices

#### 3.4.1. Dry Biomass Determination

Compound **1** dissolved in DMSO (0.5 mL) was added to four 2 L Erlenmeyer flasks containing Sabouraud medium, resulting in final concentrations of 25, 50, 75, and 100 µg/mL. Fluconazole (**8**) was tested in the flask at a concentration of 50 µg/mL. The positive control contained medium with the addition of DMSO (0.5 mL) without antifungals. The inoculum of *R. mucilaginosa* IHEM 18459 with an OD_600_ = 0.180 and an amount equal to 10% was added to the Sabouraud medium, resulting in a total volume of 500 mL. After 7 days of incubation at 25 °C, the biomass was washed three times with distilled water to remove the medium and centrifuged at 3220 RCF for 15 min at 8 °C. The biomass was then frozen and lyophilized to a constant weight.

#### 3.4.2. Plate Count Method

The fungicidal activity of 3-*n*-butylphthalide (**1**) was visualized using the plate count method. Preincubation cultures of *R. mucilaginosa* IHEM 18459 were prepared in flasks with Sabouraud medium. First, various concentrations of 3-*n*-butylidenephthalide (**1**) were added to DMSO (0.5 mL) in each flask. Fluconazole (**8**) was administered as a single dose. Subsequently, 1 mL of the inoculum at a density of 1 × 10^3^–5 × 10^3^ CFU/mL was added to the samples. Flasks were incubated on a rotary shaker at 30 °C for 48 h. Serial dilutions of each sample were then prepared, and 100 µL of cultures were transferred to agar plates and spread on the surface. After further incubation for 48 h at 30 °C, the yeast colonies were counted.

Antimicrobial activity assessed in food matrices: 25 g of commercially available yogurt was placed in sterile Nasco Whirl-Pak bags. 3-*n*-butylidenephthalide (**1**) was tested at the final concentrations of 50, 150, 250, 350, 450, and 550 µg/mL in DMSO (0.5 mL). The control involved only solvent addition. Subsequently, 1 mL of inoculum of *R. mucilaginosa* at a density of 1 × 10^3^–5 × 10^3^ CFU/mL was added to each bag. After their incubation at 30 °C for 48 h, serial dilutions of each sample were performed. Selected dilutions (100 µL) were spread onto the surface of Sabouraud agar using a cell spreader. The tests were performed in triplicate. After 48 h incubation at 30 °C, the yeast colonies were counted.

### 3.5. Microbial Transformation of 3-n-Butylidenephthalide *(**1**)* by R. mucilaginosa IHEM 18459

First, 1 mL of 0.5 McFarland inoculum of *R. mucilaginosa* IHEM 18459 was added to the Sabouraud medium, resulting in a total volume of 75 mL. After eight days of incubation on a rotary shaker at 25 °C, 20 mg of 3-*n*-butylidenephthalide (**1**) in 0.5 mL of acetone was added. Transformations were continued for an additional 8 days. The culture was then centrifuged at 3220 RCF for 15 min, and the yeast cells were separated from the supernatant. Mycelia and supernatants were extracted separately using 25 mL of ethyl acetate. The extracts were subjected to analysis using HPLC-DAD. The mobile phase was 0.5% formic acid (A) and acetonitrile (B). Gradient elution conditions were as follows: 0–3 min, 65% A/35% B; 3–12 min, 35% A/65% B; 12–13 min, 10% A/90% B; 13–14 min, 0% A/100% B; 14–16 min 0% A/100% B; 16–19 min, 65% A/35% B; and 19–23 min, 65% A/35% B. The following parameters were selected: flow rate, 0.4 mL/min; column incubation temperature, 30 °C; and detection wavelength, 275 nm.

### 3.6. The Influence of 3-n-Butylidenephthalide *(**1**)* on the ROS Level

The level of oxidative stress was assessed using the adherent cell microplate assay. Briefly, 100 µL of *R. mucilaginosa* inoculum (OD_600_ = 0.1) in PBS was added to the wells of a 96-well microtiter plate. After overnight incubation at 25 °C and adhesion of the cells, PBS was collected from above the cell sediment. Then, 100 µL of 40 µM H_2_DCFDA was added to each well of the first four rows, and 100 µL of PB buffer was added to the next four rows (unstained background control). After incubation of the plate at 37 °C for 45 min, the dye and PBS were removed from the wells. The cells were washed with 10× diluted PBS, followed by the addition of 100 µL of the same buffer. Next, 100 µL of compound **1** at 25, 50, 75, and 100 µg/mL and fluconazole (**8**) at a concentration of 50 µg/mL were loaded into wells and mixed. The positive control contained 3% H_2_O_2_, whereas the negative control did not contain the inhibitor. After 4 h of incubation at 25 °C, fluorometric measure was taken at excitation and emission wavelengths of 485 nm and 385 nm, respectively. 

### 3.7. Confocal Microscopy

The 24 h yeast inoculum was standardized in Sabouraud medium to obtain a density of 0.5 McFarland and transferred to a sterile tube. After centrifugation, the medium was discarded, and the precipitate was suspended in PBS. The sample was then transferred to a Millicell^®^ EZ SLIDE chamber and assembled on a microscope slide. Next, 20 µL of 3-*n*-butylidenephthalide (**1**), at a final concentration of 100 µg/mL, was added to the wells and mixed. After 20 min, the slide was dismounted, and the cells were stained with Calcofluor White, according to the manufacturer’s recommendations.

### 3.8. Synergistic Effect—Determination of Fractional Inhibitory Concentration (FIC)

To test the synergistic effect of the compounds, a checkerboard method was performed based on the methods described by Pillai et al. [42] and Krężel et al. [15]. Sabouraud medium was used for all tests. First, 3-*n*-butylidenephthalide (**1**) and fluconazole (**8**) were prepared in a DMSO solution and then diluted directly in the medium in a 96-well microtiter plate to obtain final concentrations of 1–528 μg/mL for fluconazole (**8**) and 0.5–32 μg/mL for 3-*n*-butylidenephthalide (**1**). The reference sample was diluted in DMSO with Sabouraud medium without the inoculum. Samples containing only one of the tested compounds served as controls. All the samples were tested in triplicate. The plates were then incubated separately in a thermoshaker at 25 °C, using 1000 RPM agitation. The absorbance was read at a wavelength of 595 nm to determine the antifungal activity. A synergistic effect was recorded when the inhibition caused by the mixture of both compounds was higher than that of the separately tested agents. FIC was calculated as follows:

$$\sum FIC=\frac{MIC\;of\;agent\;A\;in\;combination}{MIC\;of\;agent\;A\;tested\;separately}+\frac{MIC\;of\;agent\;B\;in\;combination}{MIC\;of\;agent\;B\;tested\;separately}$$

### 3.9. The Determination of Carotenoids in Biomass

#### 3.9.1. Samples Preparation

Because of carotenoid instability, the subsequent cultures were grown to obtain fresh samples. The growth conditions were analogous to those of the previous experiment with the determination of dry biomass; however, at a low scale, the total volume was 50 mL. After seven days of incubation at 25 °C, the biomass was washed three times with distilled water and frozen.

Subsequently, washed yeast cells (0.5 g) were defrosted, dissolved in 3 mL PBS, pH 7.2, and disintegrated using an ultrasonic homogenizer for 1200 s (using an 80 s:20 s work–rest cycle). The lysates were centrifuged for 3 min and the supernatants were discarded. Samples were extracted three times (two times with DMSO and once with Folch solution) using the following procedure: vortexing for 5 min at 1600 RPM with 2 mL of the solvent and centrifugation for 3 min. The fractions were collected, and the Folch solution was evaporated using a rotary evaporator. Subsequently, 1 mL of the remaining mixture was evaporated under a stream of nitrogen. The residue was dissolved in gradient-grade acetonitrile and subjected to HPLC and HR-APCI–MS/MS analyses.

#### 3.9.2. HR-APCI–MS/MS Analyses

Pigments determination and quantitative comparative analysis: The pigments were eluted with A-water with 0.1% formic acid, B-acetonitrile with 0.1% formic acid, and C-isopropanol with 0.1% formic acid in gradient mode: 0 min, 25% A/25% B/50% C; 0–45 min, 2.5% A/2.5% B/95% C; 45–50 min, 2.5% A/2.5% B/95% C; and 51–60 min, 25% A/25% B/50% C.

The flow rate was 200 µL/min. The operating parameters were as follows: nebulizer pressure: 0.3 bar, heating gas flow: 3.0 L/min, heating gas temperature, 250 °C; data acquisition range, *m*/*z* 50–2000 *m*/*z*; ionization mode, positive; and ion source energy, 10 eV.

#### 3.9.3. HPLC-DAD Analyses of β-Carotene Content

To determine the *β*-carotene calibration curve, the results were calculated according to the dry mass. The mobile phase comprised water (A) and acetonitrile (B). Gradient elution conditions were as follows: 0–2 min, 10% A/0% B; 2–10 min, 0% A/100% B; 10–20 min, 0%A/100% B; 20–22 min, 10% A/90% B; and 22–27 min, 10% A/90% B. The following parameters were selected: flow rate, 0.9 mL/min; column incubation temperature, 30 °C; and detection wavelength, 450 nm.

### 3.10. FAME Content

FAME content was determined using 50 mg of dry yeast biomass. The biomass was heated under reflux using 5 mL of 0.5 M KOH in methanol for 15 min. Next, 2 mL of boron trifluoride in diethyl ether was added, and the mixture was heated for 5 min. After cooling, the mixture was extracted with hexane (1 mL). Samples were analyzed using the following temperature program: 100 °C (hold for 3 min), 240 °C (2.5 °C/min; hold for 4 min), 260 °C (15 °C/min; hold for 1 min). FAMEs were detected by comparing their retention times with those of the standard.

### 3.11. Ergosterol Content

Ergosterol was determined using the method described by Arthington-Skaggs et al. [70] with minor modifications, using wet biomass previously obtained for carotenoid determination. Briefly, wet biomass samples were weighted and subjected to saponification with 25% potassium hydroxide in ethanol. After 1 h incubation at 85 °C and cooling, samples were extracted with hexane using a vortex, and the upper layer was collected, diluted 5× in ethanol, and measured using a spectrophotometer scanning between 200 and 300 nm. The ergosterol content was calculated as the percentage of the dry biomass.

## 4. Conclusions

Synthetic phthalide lactones limit the growth of *R. mucilaginosa* IHEM 18459 cells. Lactone **1** demonstrated a synergistic effect with fluconazole (**8**), which reduced the amount of this drug required for yeast inhibition.

In this study, we performed various experiments to determine the effects of phthalide lactone on *R. mucilaginosa* IHEM 18459 cells. We have shown that 3-*n*-butylidenephthalide (**1**) is not metabolized to other compounds and accumulates in yeast cells, which contributes to an increase in ROS. The effect of 3-*n*-butylidenephthalide (**1**) on *R. mucilaginosa* IHEM 18459 cells was similar to that of *C. albicans* ATCC 10231, *C. albicans* ATCC 2091, and *C. guilliermondii* KKP 3390 cells, confirming the mechanism proposed in the literature.

The data show the strong influence of phthalide **1** on the quantitative composition of FAME, where, for example, at a concentration of 75 mg/mL, it decreased three times. The increasing proportion of unsaturated fatty acids suggested that the fluidity of the cell membrane increased. This effect may be related to the expression of the genes encoding fatty acid desaturases. 3-*n*-Butylidenephthalide (**1**) did not notably change the amount of ergosterol. This means that the observed fungistatic activity of lactones **1**–**7** and fluconazole (**8**) is not related to cell membrane damage associated with the lack of ergosterol.

Cultures of *R. mucilaginosa* IHEM 18459 grown in the presence of 3-*n*-butylidenephthalide (**1**) changed color from orange/red to light yellow, which was related to the inhibition of torulene and torularhodin. 

In some experiments, we observed a surprising effect of fluconazole (**8**): an increase in the production of *β*-carotene, torulene, and torularhodin, or an increase in ROS. The high value of IC_50_ and IC_90_ and the lack of substantial effect on ergosterol content may be related to the fluconazole resistance of *R. mucilaginosa* IHEM 18459.

## Figures and Tables

**Table 1:** Fungistatic activity (IC_50_) using microdilution method, in silico prediction of lipophilicity (Log P), solubility (log S), and oral toxicity to rat cells (LC_50_). Prediction of CYP isoenzymes inhibition: CYP1A2, CYP2C19, CYP2C9, CYP2D6, and CYP3A4, based on chemical structures of compounds **1**–**8**.

Compound	IC_50_ at 25 °C [µg/mL]	IC_50_ at 30 °C [µg/mL]	Log P	Log S	LC_50_ [mg/kg]	Inhibition of:
**1**	13	13	2.93	−3.30	1865	CYP1A2
**2**	20	23	2.81	−3.01	4872	CYP1A2 CYP2C9
**3**	38	32	2.78	−2.43	1895	CYP1A2
**4**	130	109	2.44	−2.45	4061	CYP1A2
**5**	65	66	2.93	−3.20	5184	-
**6**	42	43	2.96	−3.79	4702	CYP2C9
**7**	54	57	2.93	−3.20	4186	CYP2C9
**8**	199	207	0.88	−1.63	584	CYP2C19

**Table 2:** *R. mucilaginosa* IHEM 18459 pigments detected using APCI-LC-HR-MS and their relative composition depending on the different concentrations of the 3-*n*-butylidenephthalide (**1**) and fluconazole (**8**) [µg/mL].

Carotenoid	APCI-LC-HR-MS [M + H]^+^	0	25	50	75	100	F50
Phytoene	545.5081	1	50.92	70.61	95.83	35.33	3.52
Neurosporene	539.4611	n.d.	n.d.	n.d.	d.	n.d.	n.d.
*β*-carotene + lycopene	537.4445	1	4.45 ^a^	1.10 ^a^	1.72 ^a^	0.20	2.30 ^a^
Torulene	535.4280	1	0.88	0.21	0.35	0.07	1.66
Torularhodin	565.4063	1	0.41	0.01	0.05	n.d.	1.63

F means fluconazole; ^a^ the identification of these pigments was confirmed by the compounds’ standards using HPLC-DAD; n.d.—not detected; and d.—detected.

**Table 3:** *β*-Carotene content in the *R. mucilaginosa* IHEM 18459 biomass depending on the different concentrations of the 3-*n*-butylidenephthalide (**1**) and fluconazole (**8**); data represent the mean  ±  standard deviation.

Concentration of Compounds [µg/mL]	*β*-Carotene Content [mg/g d.m.]
0	0.2108 ± 0.0055
25	0.4433 ± 0.0282
50	0.2305 ± 0.0108
75	0.0262 ± 0.0042
100	n.d.
F50	0.4792 ± 0.0055

F means fluconazole.

**Table 4:** The impact of the dose of the compounds on the profile of all detected FAMEs; the data are shown as the mean ± standard deviation.

Concentration of a Compound [µg/mL]	Saturated [%]	Unsaturated [%]	Unsaturated Including:	
			Monounsaturated (MUFA) [%]	Polyunsaturated (PUFA) [%]
0	19.02 ± 0.08	80.98 ± 0.08	98.16 ± 0.14	1.84 ± 0.14
25	20.31 ± 0.05	79.69 ± 0.05	95.06 ± 0.02	4.94 ± 0.02
50	16.77 ± 0.01	83.23 ± 0.01	95.74 ± 0.02	4.26 ± 0.02
75	16.54 ± 0.04	83.46 ± 0.04	96.43 ± 0.09	3.57 ± 0.09
100	14.12 ± 0.18	85.88 ± 0.18	95.82 ± 0.05	4.18 ± 0.05
F50	15.27 ± 0.14	84.73 ± 0.14	98.00 ± 0.12	2.00 ± 0.12

F means fluconazole.

## Data Availability

The data presented in this study are available on request from the corresponding author.

## Supplementary Materials

The following supporting information can be downloaded at: https://www.mdpi.com/article/10.3390/molecules28145423/s1, Figure S1a: ^1^H NMR of 3-*n*-butylidenephthalide (**1**); Figure S1b: ^13^C NMR of 3-*n*-butylidenephthalide (**1**); Figure S1c: HR-ESI-MS/MS of 3-*n*-butylidenephthalide (**1**); Figure S2a: ^1^H NMR of 3-*n*-butylphthalide (**2**); Figure S2b: ^13^C NMR of 3-*n*-butylphthalide (**2**); Figure S2c: HR-ESI-MS/MS of 3-*n*-butylphthalide (**2**); Figure S3: HR-ESI-MS/MS of 3-*n*-propylidenephthalide (**3**); Figure S4a: ^1^H NMR of 3-*n*-propylphthalide (**4**); Figure S4b: ^13^C NMR of 3-*n*-propylphthalide (**4**); Figure S4c: HR-ESI-MS/MS of 3-*n*-propylphthalide (**4**); Figure S5a: ^1^H NMR of 3-butyl-4,5,6,7-tetrahydrophthalide (**5**); Figure S5b: ^13^C NMR of 3-Butyl-4,5,6,7-tetrahydrophthalide (**5**); Figure S5c: HR-ESI-MS/MS of 3-Butyl-4,5,6,7-tetrahydrophthalide (**5**); Figure S6a: ^1^H NMR of 3-*n*-butyl-hexahydrophthalide (**6**); Figure S6b: ^13^C NMR of 3-*n*-butyl-hexahydrophthalide (**6**); Figure S6c: HR-ESI-MS/MS of 3-*n*-butyl-hexahydrophthalide (**6**); Figure S7a: ^1^H NMR of 3-*n*-butyl-1,2,6,7-tetrahydrophthalide (**7**); Figure S7b: b ^13^C NMR of 3-*n*-butyl-1,2,6,7-tetrahydrophthalide (**7**); Figure S7c: HR-ESI-MS/MS of 3-*n*-Butyl-1,2,6,7-tetrahydrophthalide (**7**).

## Sample Availability

Samples of the compounds are available from the authors upon request.
