# Supplementary material for: Fungistatic Effect of Phthalide Lactones on Rhodotorula mucilaginosa
Source: Molecules. 2023 Jul 15;28(14):5423. doi: 10.3390/molecules28145423 (PMC10384090; doi:10.3390/molecules28145423)
Supplement: Supplementary file 1 [file molecules-28-05423-s001.zip › molecules-2459075-supplementary.pdf]

molecules

Fungistatic effect of phthalide lactones on *Rhodotorula mucilaginosa*

Joanna Gach[1]*, Teresa Olejniczak[1]*, Jakub Pannek[1] and Filip Boratyński[1]

[1] Department of Food Chemistry and Biocatalysis, Wrocław University of Environmental and Life Sciences, Norwida 25, 50-375 Wrocław, Poland; jpannek@gmail.com (J.P.), filip.boratynski@upwr.edu.pl (F.B.)
* Correspondence: joanna.gach@upwr.edu.pl (J.G.), teresa.olejniczak@upwr.edu.pl (T.O.)

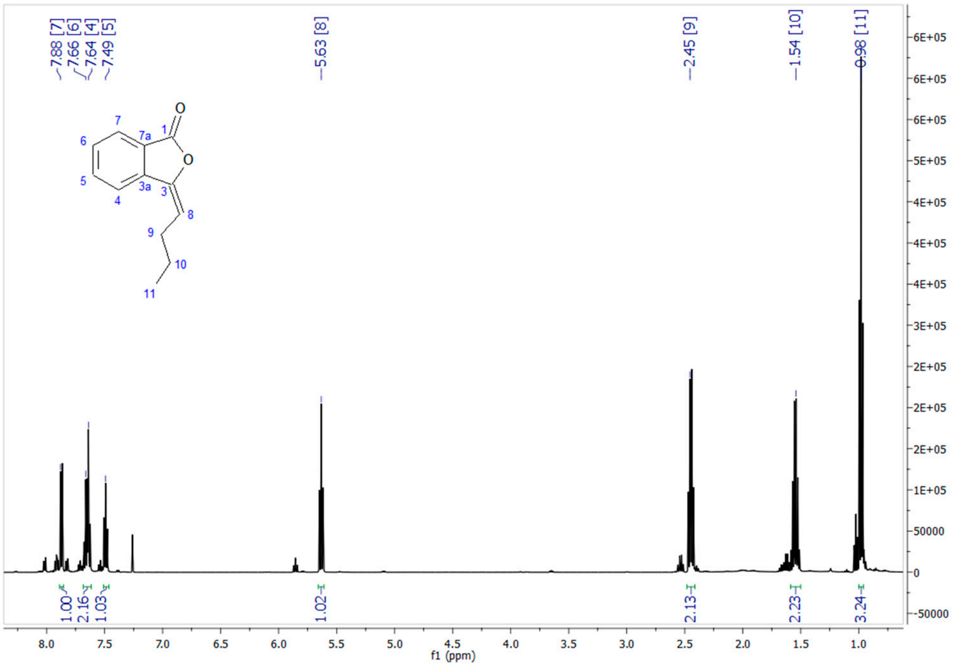

Figure S1a. ^{1}H NMR of 3-*n*-butylidenephthalide (**1**).

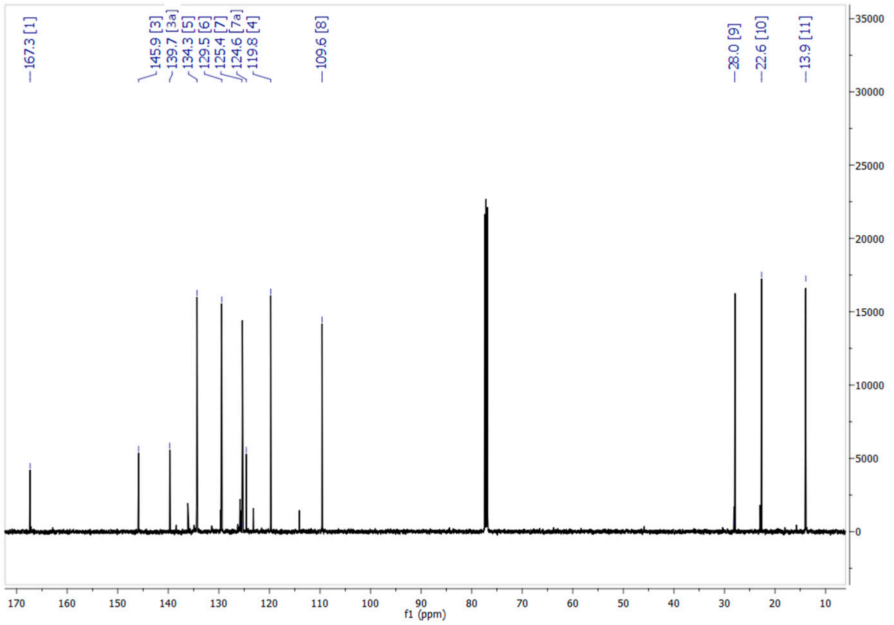

Figure S1b. ^{13}C NMR of 3-*n*-butylidenephthalide (**1**).

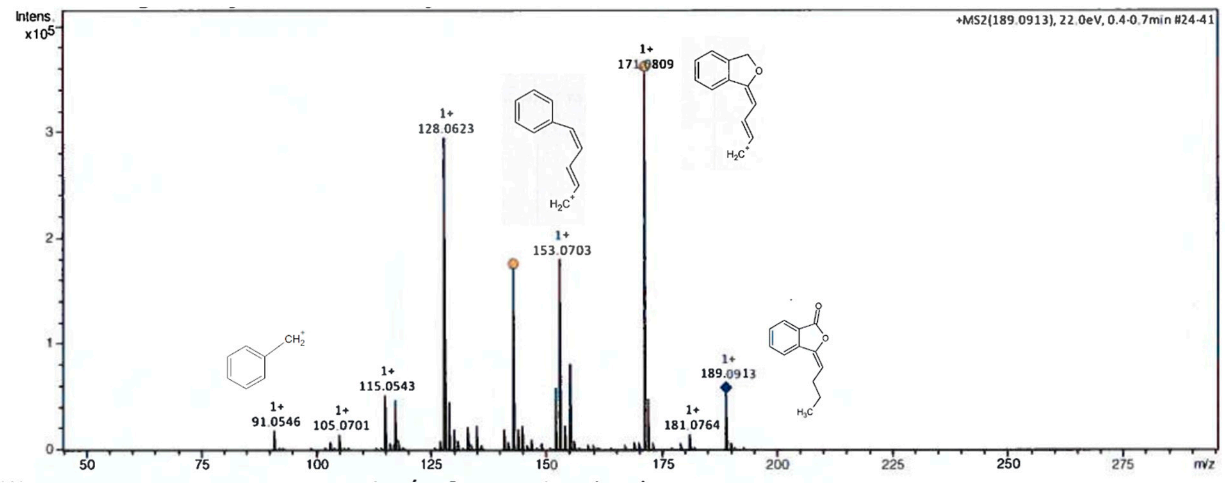

Figure S1c. HR-ESI-MS/MS of 3-*n*-butylidenephthalide (**1**).

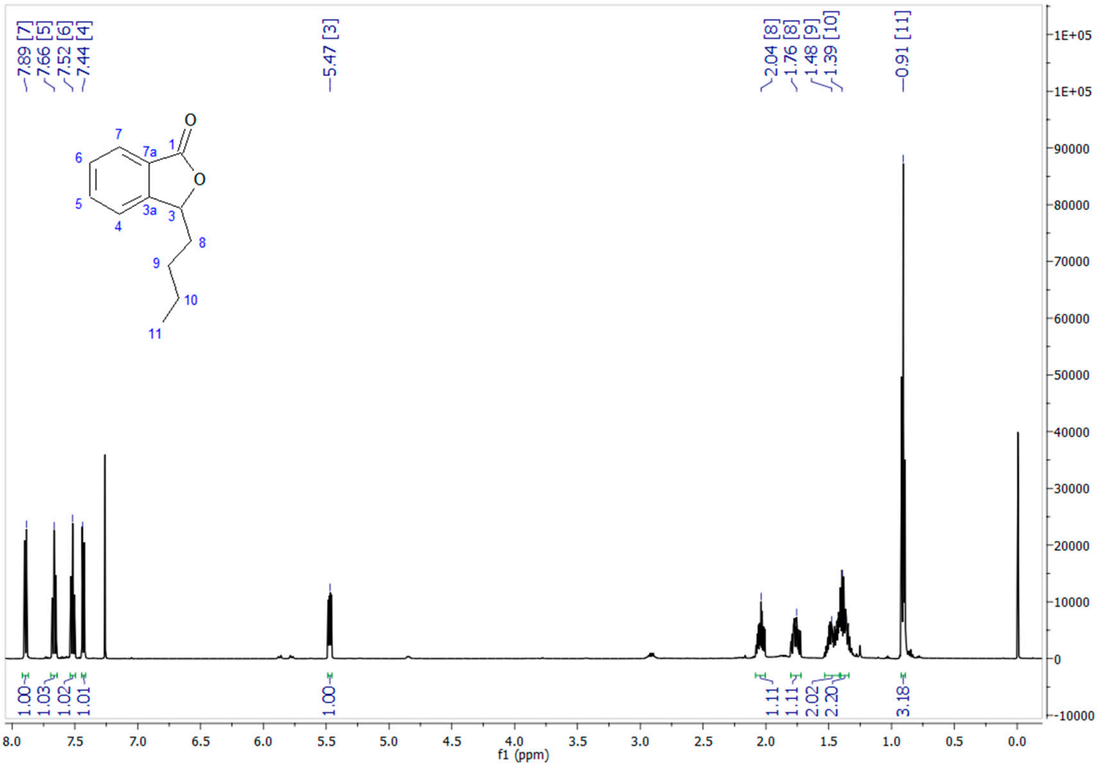

Figure S2a. ^{1}H NMR of 3-*n*-butylphthalide (**2**).

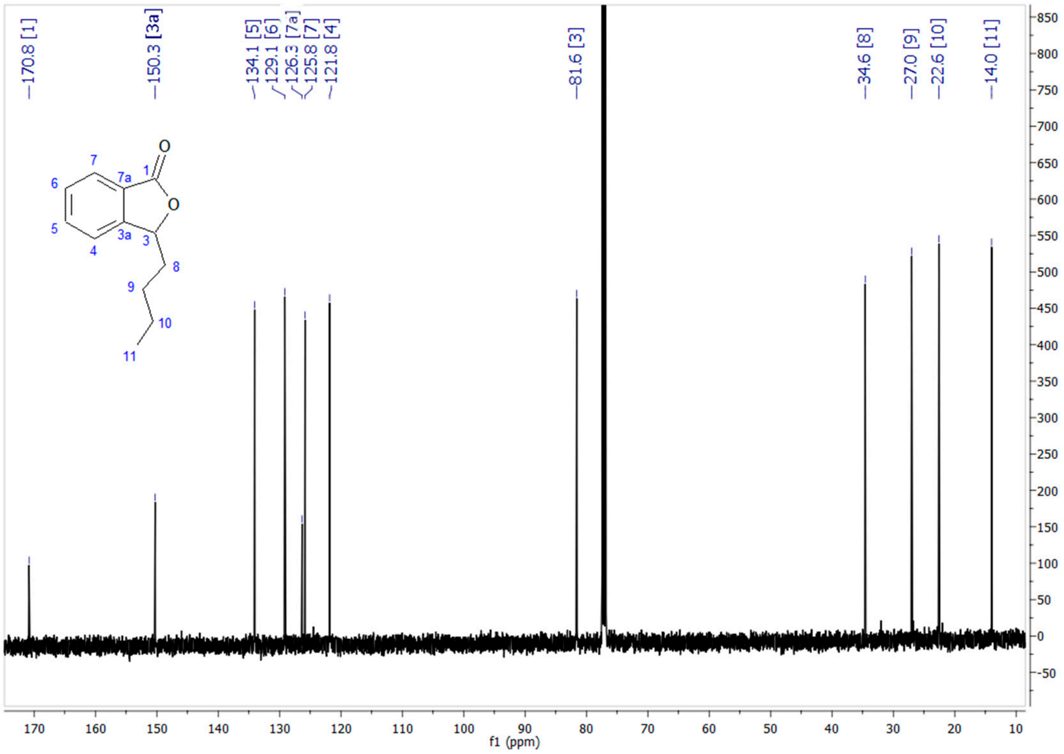

Figure S2b. ^{13}C NMR of 3-*n*-butylphthalide (**2**).

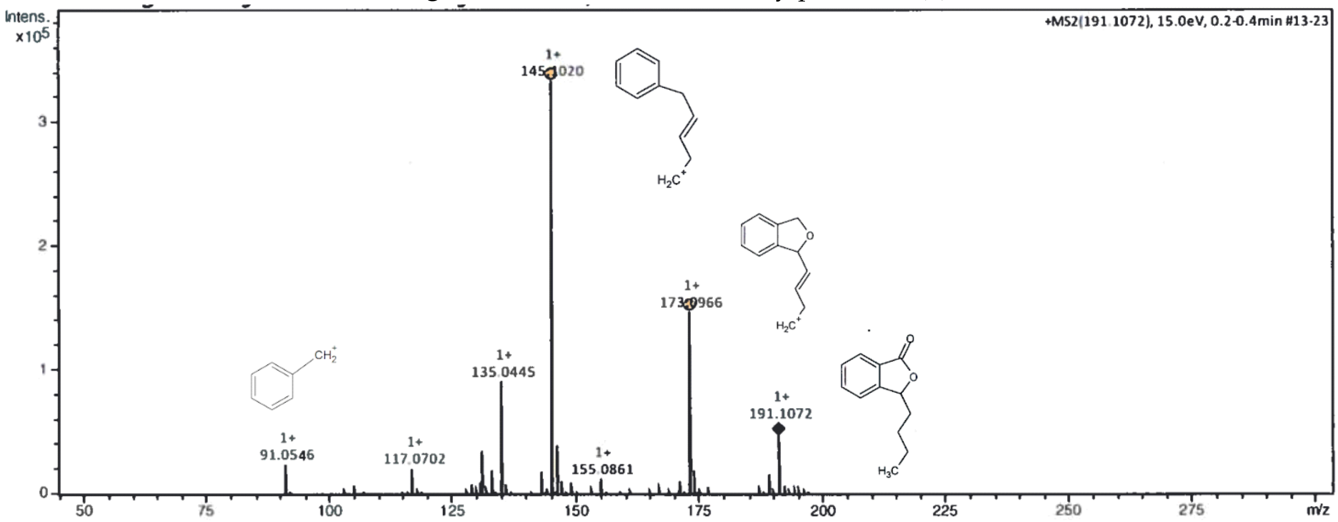

Figure S2c HR-ESI-MS/MS of 3-*n*-butylphthalide (**2**).

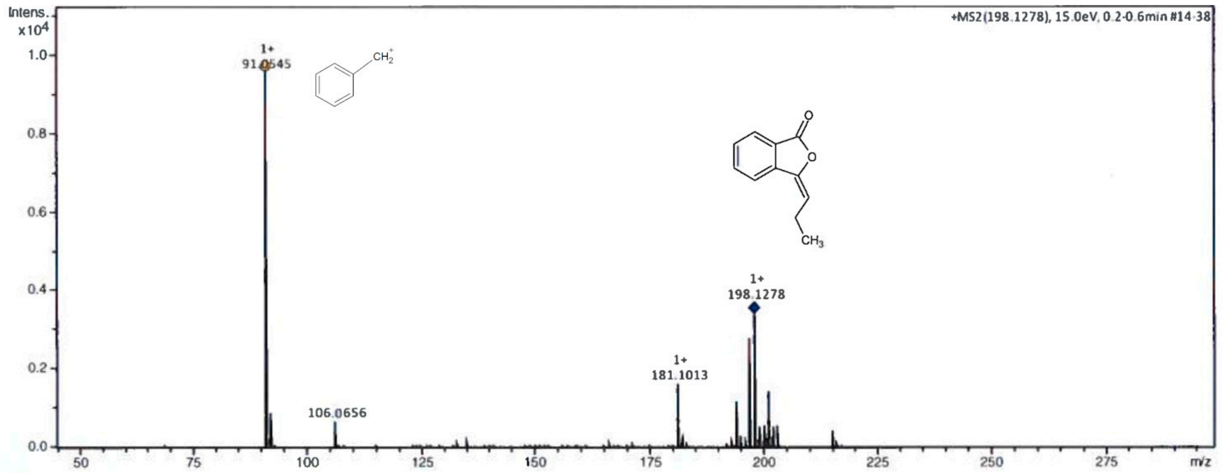

Figure S3 HR-ESI-MS/MS of 3-*n*-propylidenephthalide (**3**).

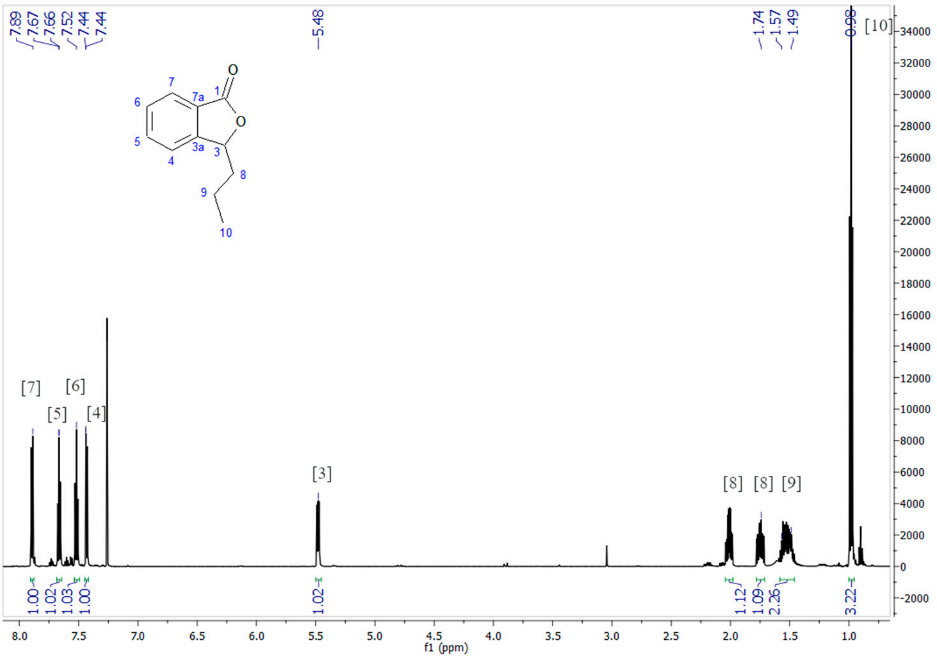

Figure S4a ¹H NMR of 3-*n*-propylphthalide (**4**).

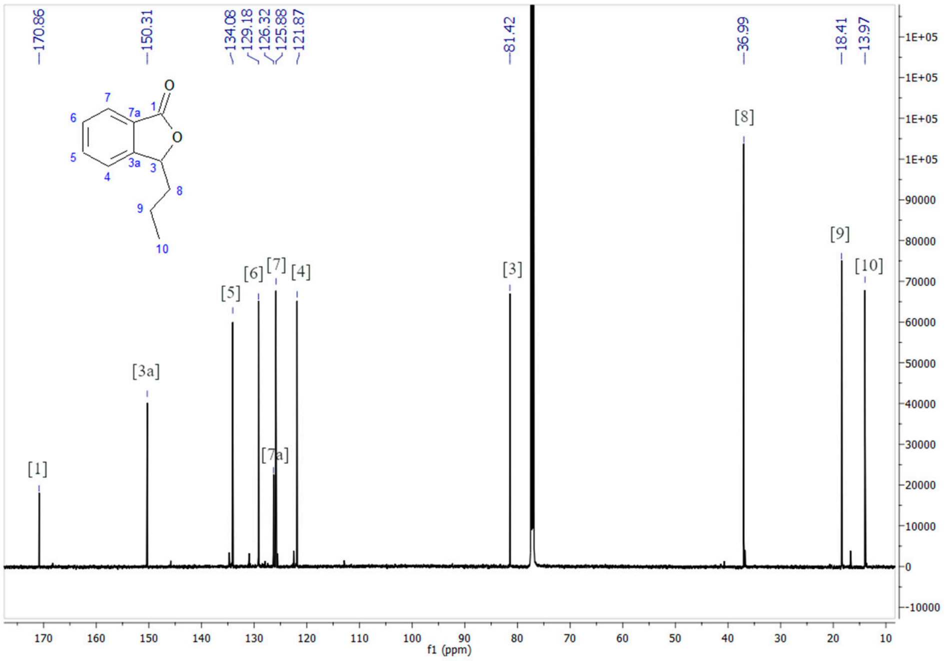

Figure S4b ¹³C NMR of 3-*n*-propylphthalide (**4**).

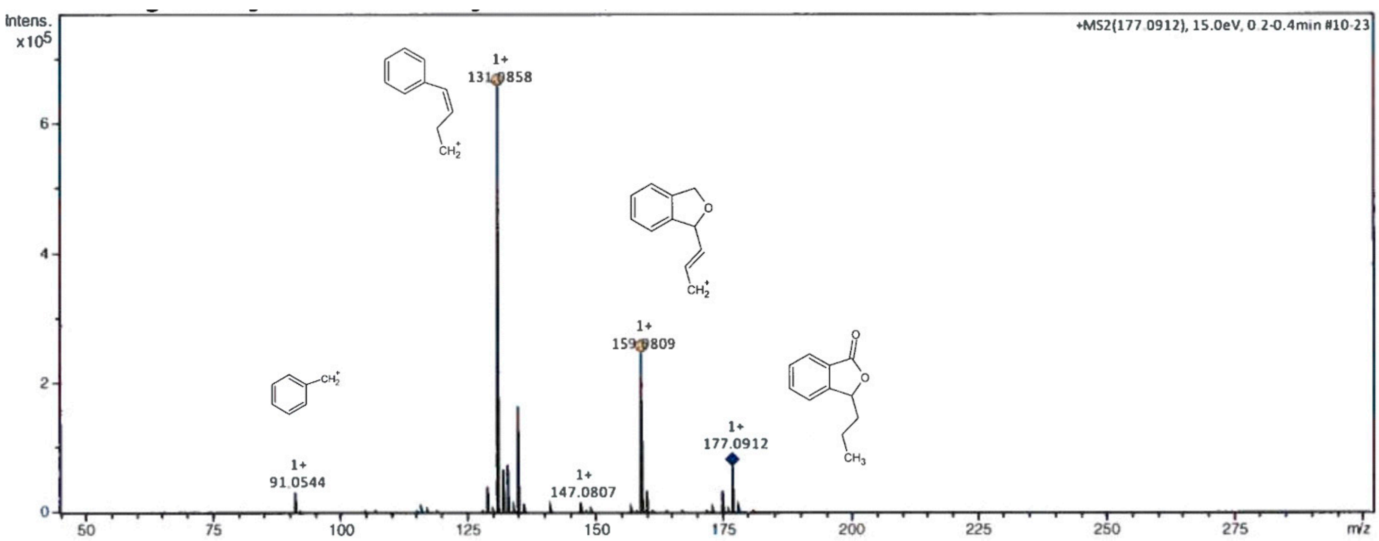

Figure S4c HR-ESI-MS/MS of 3-*n*-propylphthalide (**4**).

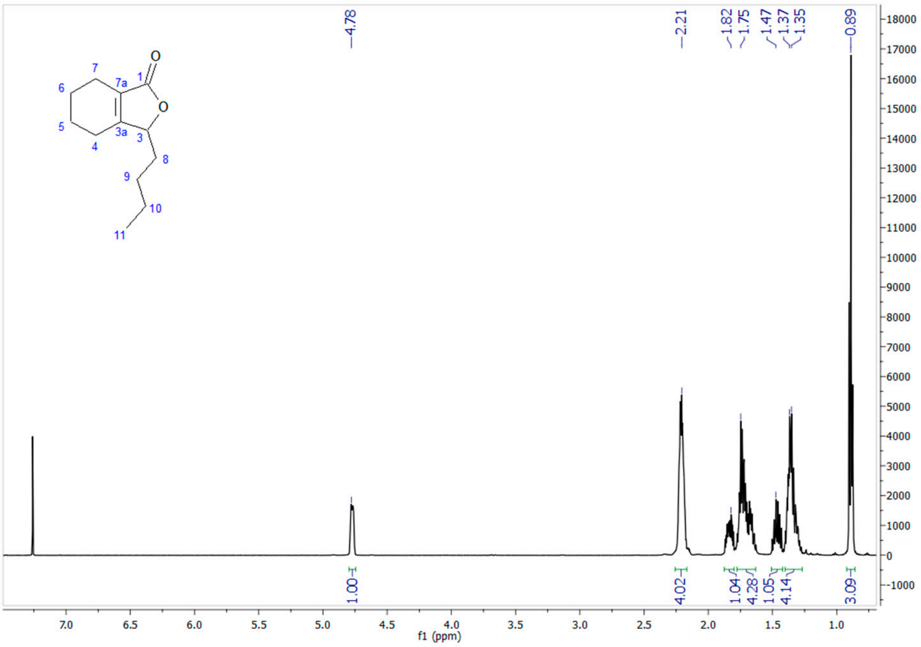

Figure S5a. ^{1}H NMR of 3-butyl-4,5,6,7-tetrahydrophthalide (**5**).

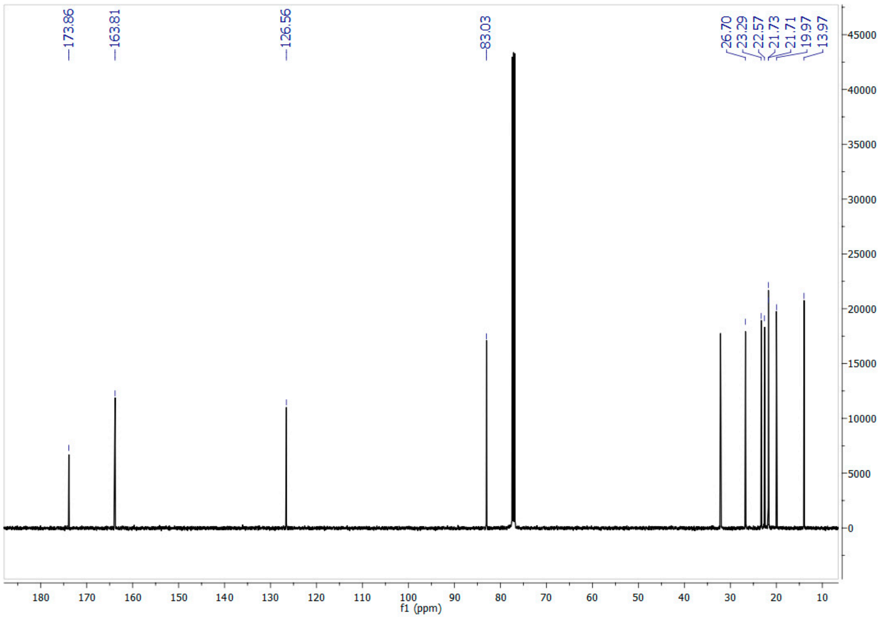

Figure S5b. ^{13}C NMR of 3-Butyl-4,5,6,7-tetrahydrophthalide **(5).**

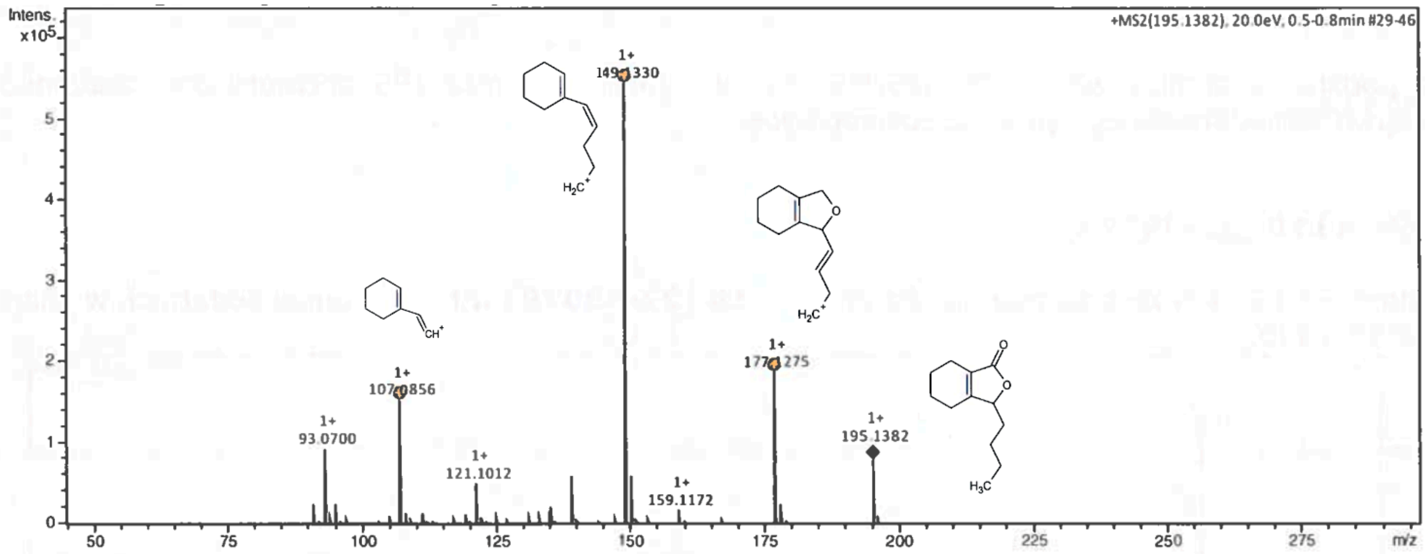

Figure S5c. HR-ESI-MS/MS of 3-Butyl-4,5,6,7-tetrahydrophthalide **(5).**

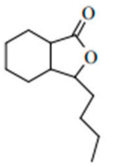

6

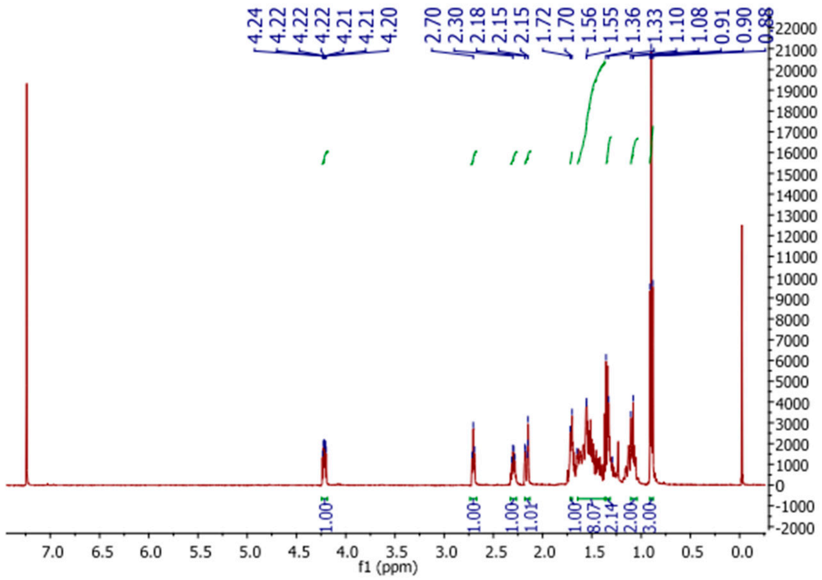

Figure S6a. ^{1}H NMR of 3-*n*-butyl-hexahydrophthalide (**6**).

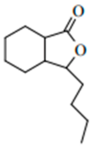

6

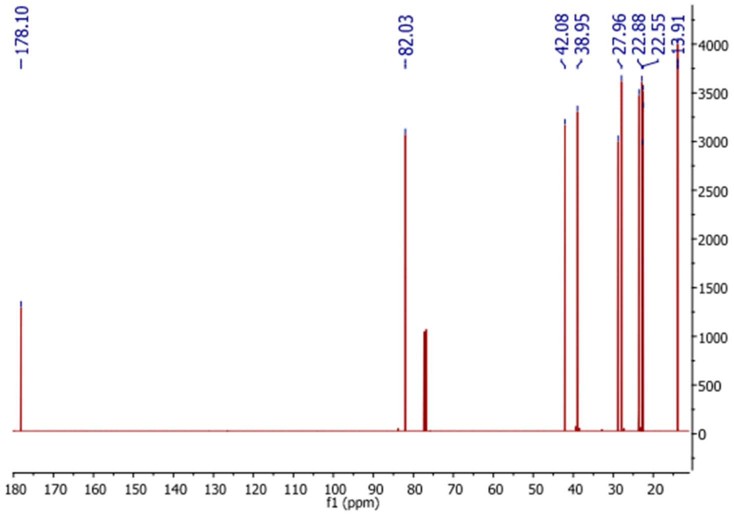

Figure S6b. b ^{13}C NMR of 3-*n*-butyl-hexahydrophthalide (**6**).

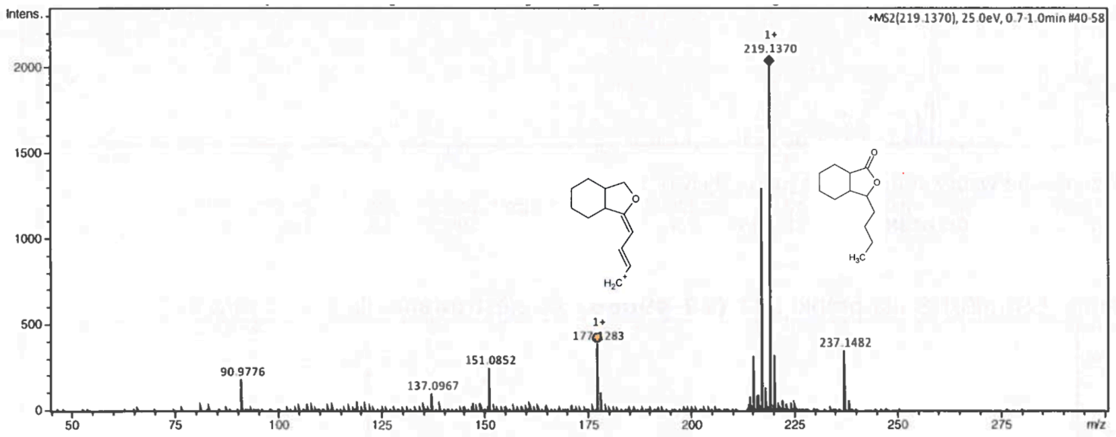

Figure S6c. HR-ESI-MS/MS of 3-*n*-butyl-hexahydrophthalide (**6**).

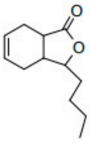

7

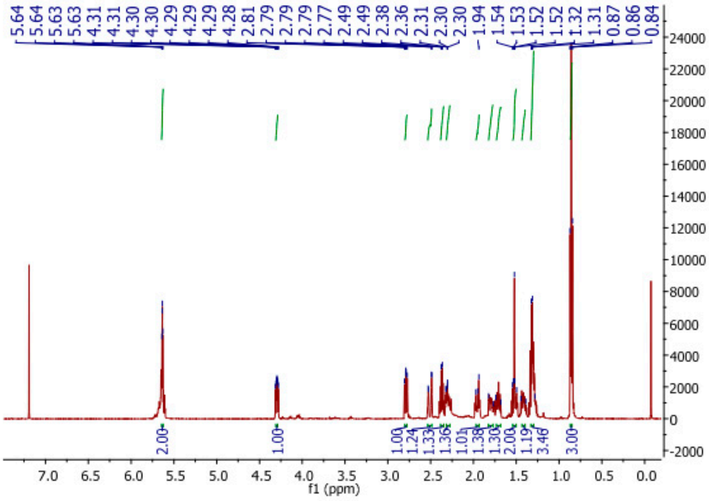

Figure S7a. ^{1}H NMR of 3-*n*-butyl-1,2,6,7-tetrahydrophthalide (**7**).

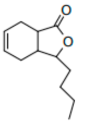

7

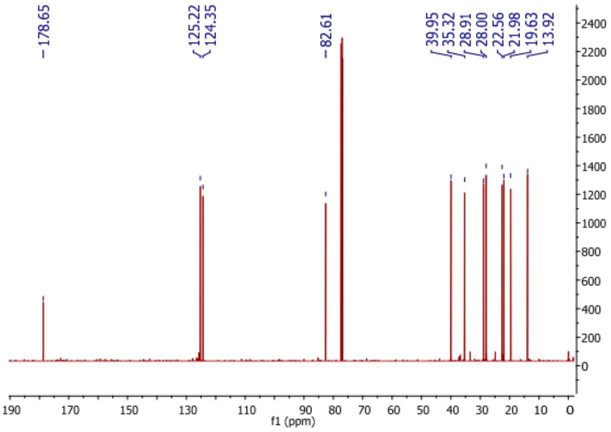

Figure S7b. b ^{13}C NMR of 3-*n*-butyl-1,2,6,7-tetrahydrophthalide **(7).**

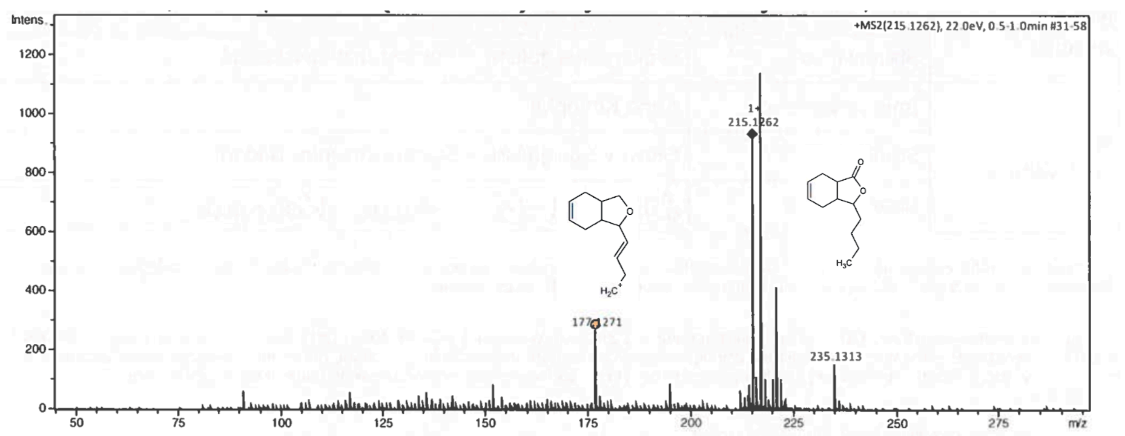

Figure S7c. HR-ESI-MS/MS of 3-*n*-Butyl-1,2,6,7-tetrahydrophthalide **(7).**
